# Bias at the third nucleotide of codon pairs in virus and host genomes

**DOI:** 10.1038/s41598-022-08570-w

**Published:** 2022-03-16

**Authors:** Ewan P. Plant, Zhiping Ye

**Affiliations:** Laboratory of Pediatric and Respiratory Viral Disease, Office of Vaccine Research and Review, CBER, FDA, Silver Spring, MD USA

**Keywords:** Computational biology and bioinformatics, Evolution, Genetics, Microbiology, Molecular biology

## Abstract

Genomes of different sizes and complexity can be compared using common features. Most genomes contain open reading frames, and most genomes use the same genetic code. Redundancy in the genetic code means that different biases in the third nucleotide position of a codon exist in different genomes. However, the nucleotide composition of viruses can be quite different from host nucleotide composition making it difficult to assess the relevance of these biases. Here we show that grouping codons of a codon-pair according to the GC content of the first two nucleotide positions of each codon reveals patterns in nucleotide usage at the third position of the 1st codon. Differences between the observed and expected biases occur predominantly when the first two nucleotides of the 2nd codon are both S (strong, G or C) or both W (weak, A or T), not a mixture of strong and weak. The data indicates that some codon pairs are preferred because of the strength of the interactions between the codon and anticodon, the adjacent tRNAs and the ribosome. Using base-pairing strength and third position bias facilitates the comparison of genomes of different size and nucleotide composition and reveals patterns not previously described.

## Introduction

There are differences between viral genomes and the genomes of host cells. Cellular organisms have double-stranded DNA genomes, but viral genomes can be DNA or RNA, single-stranded or double-stranded. Viral genomes are smaller than host genomes and regulatory signals often overlap with open reading frames (ORFs). Both viral and host genomes encode ORFs decoded by the same host cell protein translation machinery suggesting that there might be some genomic similarities. Many analyses comparing virus and host cell genomes have focused on nucleotide content and codon usage^1–3^. Virus codon usage does not always mimic host codon usage but there is evidence that codon usage is affected by host association^4^. More recently trends have been described for codon-pair patterns in different organisms. These analyses have revealed that codon-pair patterns are conserved between phylogenetically related organisms^5,6^. This might be due to similarities in the translational machinery (ribosomes and pool of tRNAs) and the movement of the peptidyl-tRNA with the incoming acylated tRNA or exiting tRNA deacylated-tRNA. The two subunits of the ribosome move in a ratcheting motion to pass the messager RNA (mRNA) and associated tRNAs through. The amino acid acceptor arms of the tRNAs move with the large ribosomal subunit and the anticodon arms and mRNA move with the small subunit^7,8^. Interaction between adjacent tRNAs during translation is supported by multivariate analyses of codon-pair usage and nucleotide identity in tRNA sequences^9^. Here we describe patterns in codon third nucleotide position bias in the context of codon pairs and codon:anticodon binding strength.

Ribosomes produce viral and host proteins encoded by the mRNA molecules. Amino acids are added to the peptide chain using an iterative process. The anticodon loops of tRNA molecules bind to three nucleotides of the codon specified in the mRNA and, after peptidyl transfer, the two tRNA molecules bound to the mRNA are moved through the ribosome to reposition the apparatus for the next iteration^7,8^. This process is coordinated by a network of interactions among the mRNA, tRNAs and ribosome that maintains the reading frame (three nucleotides per codon). A relationship between the network interactions and codons has been described (Supplementary Fig. 1)^10^.

There is redundancy in the genetic code and different organisms display different codon preferences. Codon preference can influence the speed of translation, protein folding and the degradation of mRNA^11^. tRNA molecules are frequently modified at the wobble position so that they can decode more than one codon. The synonymous codons that arise from this redundancy are usually grouped together in codon tables (the exceptions being codons for arginine, serine and leucine). If the first two nucleotides of a codon are adenosine (A) or thymine (T) the network interactions are ‘weak’^10^. If the first two nucleotides are guanosine (G) or cytosine (C) the network interactions are ‘strong’, and if the nucleotides are a combination then the network interactions are ‘intermediate’. Using this framework, we previously observed that codon pairs characterized by codons with weak network interactions were more frequently observed in influenza virus genomes^12^.

It is unclear whether codon-pair biases in viruses are relevant to protein production, host adaptation or genomic features, or some combination of these. The codon-pair biases can be expressed according to the under- or over-representation of observed pairs relative to the expected frequencies. This approach has been used to characterize viral genome bias with respect to the codon-pair frequencies of the human genome and to create attenuated viral genomes by recoding the genomes with codon pairs under-represented in the human genome^13^. However, there is variation in nucleotide content among different viral and host genomes^14^. More variation occurs at the third codon position than the second codon position as would be expected with the redundancy of the genetic code^14^. Codon-pair biases are influenced by both the dinucleotide spanning the codon-pair boundary and the tetranucleotide encompassing the second codon when the codon pair is positioned in the P and A sites of the ribosome^9,15^. One of the most interesting dinucleotide biases is CpG which is under-represented in vertebrate genomes and some RNA virus genomes (reviewed in^1^). The zinc-finger antiviral protein (ZAP) inhibits some of these viruses through recognition of higher levels of CpG in RNA and evasion of ZAP has been linked to the low abundance of CpG dinucleotides in an early human cytomegalovirus transcript^16^. This supports the assertion increased CpG content is the underlying reason for attenuation in recoded genomes of RNA viruses that affect humans^15^. However, studies with codon-pair deoptimization HIV genomes (controlling for CpG content) demonstrate that attenuation is associated with reduced translation efficiency^17^. Thus, the impact codon pairs have on virus viability depends on several factors including the type of virus, the position of the codon pairs within the genome, the impact on dinucleotide content, and how codon pairs affect translation^18^.

Here we compare codon-pair biases from several different host organisms and viral families to look for similarities, or differences, that might improve our understanding of codon pairs in viral genomes. We hypothesize that if protein production has a significant role then the virus and host biases will be similar because both use the same protein production apparatus. If codon-pair biases are due to virus adapting to the host then viruses from the same genera that infect divergent hosts will have biases that diverge from the expected virus genera bias and converge with the observed bias of the respective hosts. Lastly, if viral codon-pair biases are more important for maintaining genomic features then the biases are expected converge among related virus families.

**Figure 1:**
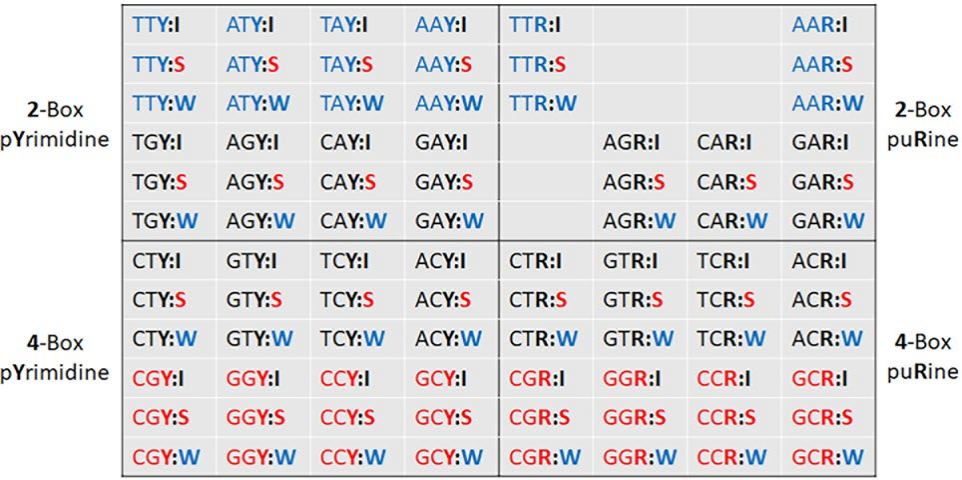
Overview of heatmap structure for assessment of third nucleotide bias in codon pairs. Codon-pairs are show as the first codon (three nucleotides) separated by a colon from the second codon type (**I**, **S** or **W** according to network interaction characteristics). The codons are colored according to network interaction characteristics: blue for ‘weak’, black for ‘intermediate’, and red for ‘strong’. Each datapoint in the heatmaps show the preferred third nucleotide for the first codon. The ratio of the third position nucleotides is shown as the greater of —(**T**/**C**) or —(**C**/**T**) when the third nucleotide is a pyrimidine (**Y**, left quadrants) and the greater of —(**A**/**G**) or —(**G**/**A**) when the third nucleotide is a purine (**R**, right quadrants) and shaded green or red respectively. 2-box codons are in the two upper quadrants and 4-box codons are in the two lower quadrants. There are no ratios for methionine, tyrosine and termination codons resulting in empty cells in the upper right quadrant.

To facilitate the comparison of codon-pair usage among viruses and hosts across a range of genera we devised a heatmap that consolidates the thousands of possible codon pairs into a 12 × 8 grid (Fig. 1). The heatmap indicates which nucleotide is preferred at the third position of the 1st codon for different combinations of ‘weak’, ‘strong’ and ‘intermediate’ codon pairs. The heatmaps can be generated for expected codon pairs using codon usage tables (CUTs), or for observed values derived from codon-pair frequency tables. Analysis of heatmaps for several organisms reveals interesting trends. The biases observed in virus and host genomes appear to converge in some examples, while in other examples they diverge. This suggests some viruses optimize protein production through codon preference while genomic characteristics hold more sway for other viruses. We discuss several examples and highlight the trends associated with different types of virus.

## Results

It has been reported that codon-pair patterns are conserved between phylogenetically related organisms but, to our knowledge, this hasn’t been investigated for viral taxa^5,6^. Frequency tables for organisms with larger genomes and more ORFs are derived from larger datasets than tables for organisms like viruses with small compact genomes. Most analyses presented here use data for virus families rather than individual species to reduce the skewing of frequency tables due to missing values. For example, the Yellow fever virus, with only 3411 codon pairs, does not have 2276 of the possible codon pairs, whereas the Flaviviridae family that Yellow fever virus belongs to has all possible codon pairs represented except for 299 codons paired with termination codons. Codon-pair frequency tables were downloaded from the CoCoPUTs database^19^ as described in the “Methods” section for several viral families (Supplementary File 1). The majority of the viral codon-pair frequency data is calculated from 10^5^–10^6^ codon pairs (Supplementary Fig. 2). The distribution of the data was assessed. The distribution graphs have long right tails indicating that some codon pairs are present higher frequencies, and the data is not normally distributed (Supplementary Fig. 2). These patterns were observed for frequency data from both viral families and higher eukaryote genomes calculated from at least 1.8^7^ codon pairs suggesting that the distribution in viral families is not a function of the size of the dataset.

**Figure 2:**
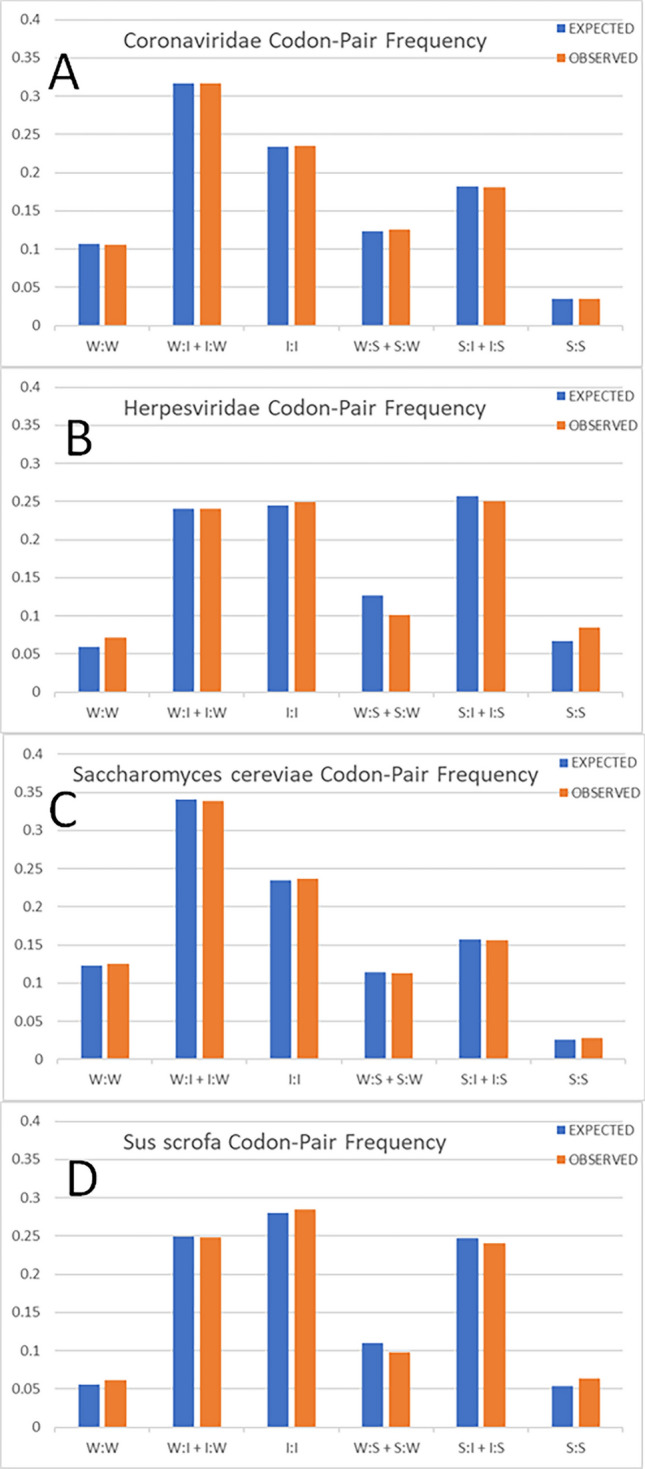
Codon-Pair Frequencies for (**A**), Coronaviridae; (**B**), Herpesviridae; (**C**), *Saccharomyces cerevisiae*; and (**D**), *Sus scrofa*. The frequency is shown on the y-axis, blue columns show the expected frequency, and orange columns show the observed frequency. The columns are grouped by network interaction strength associated with each codon: W for weak, I for intermediate, and S for strong.

To compare diverse viral and host genomes we focused on two features, bias at the third nucleotide position of the 1st codon and the GC content of the first two nucleotide positions of both codons in each codon pair. There are 4096 possible codon pairs. The codon pairs were divided into smaller groups based on the strength of the network interactions (that is, based on the GC composition of the first two nucleotides of each codon). For example, ‘weak’ codons can be paired with ‘intermediate’, ‘strong’ or ‘weak’ codons (designated W:I, W:S and W:W respectively). The expected and observed frequencies of different viral families were graphed (excluding 375 pairs containing a termination codon, Supplementary Fig. 3). The observed frequencies were generally similar to the expected frequencies amongst various genomes (Fig. 2). Genomes with lower GC content are expected to have more ‘weak’ codons and this was observed. The W:I + I:W and W:W codon pairs constitute more than 40% of the total codon pairs for Coronaviridae and *Saccharomyces cerevisiae* (which have ORFs with GC compositions of 39.5% and 39.7% respectively) whereas they constitute approximately 30% of the total codon pairs for Herpesviridae and *Sus scrofa* (which have ORFs with GC compositions of 54.4% and 52.9% respectively) (see Supplementary File 1).

The GC content of a genome does not always reflect the GC content at third codon positions (see Supplementary File 1). For example, the GC3 content for Coronaviridae (32.4%) is lower than the total GC content (39.5%) of the ORFs. In contrast, the GC3 content for Herpesviridae (61.6%) is higher than the total GC content (54.4%) of the ORFs. To better visualize the third nucleotide composition of the 1st codon in a pair we created heatmaps. The heatmaps display the more frequent purine or pyrimidine nucleotide in the third position of a codon pair. The columns on the left half of the heatmap display the bias for the 1st codons ending in a pyrimidine. The bias for 1st codons ending in a purine are displayed on the right (Fig. 1). The 1st codons are grouped according to network strength and codon box number. The 2-box groups and are displayed on the top half of the heatmap and all the 4-box groups and are displayed on the bottom. The ‘intermediate’, ‘weak’ and ‘strong’ 2nd codons are represented as separate rows when paired with ‘weak’ 2-box codons, ‘intermediate’ 2-box codons, ‘intermediate’ 4-box codons, and ‘strong’ 4-box codons (Fig. 1).

**Figure 3:**
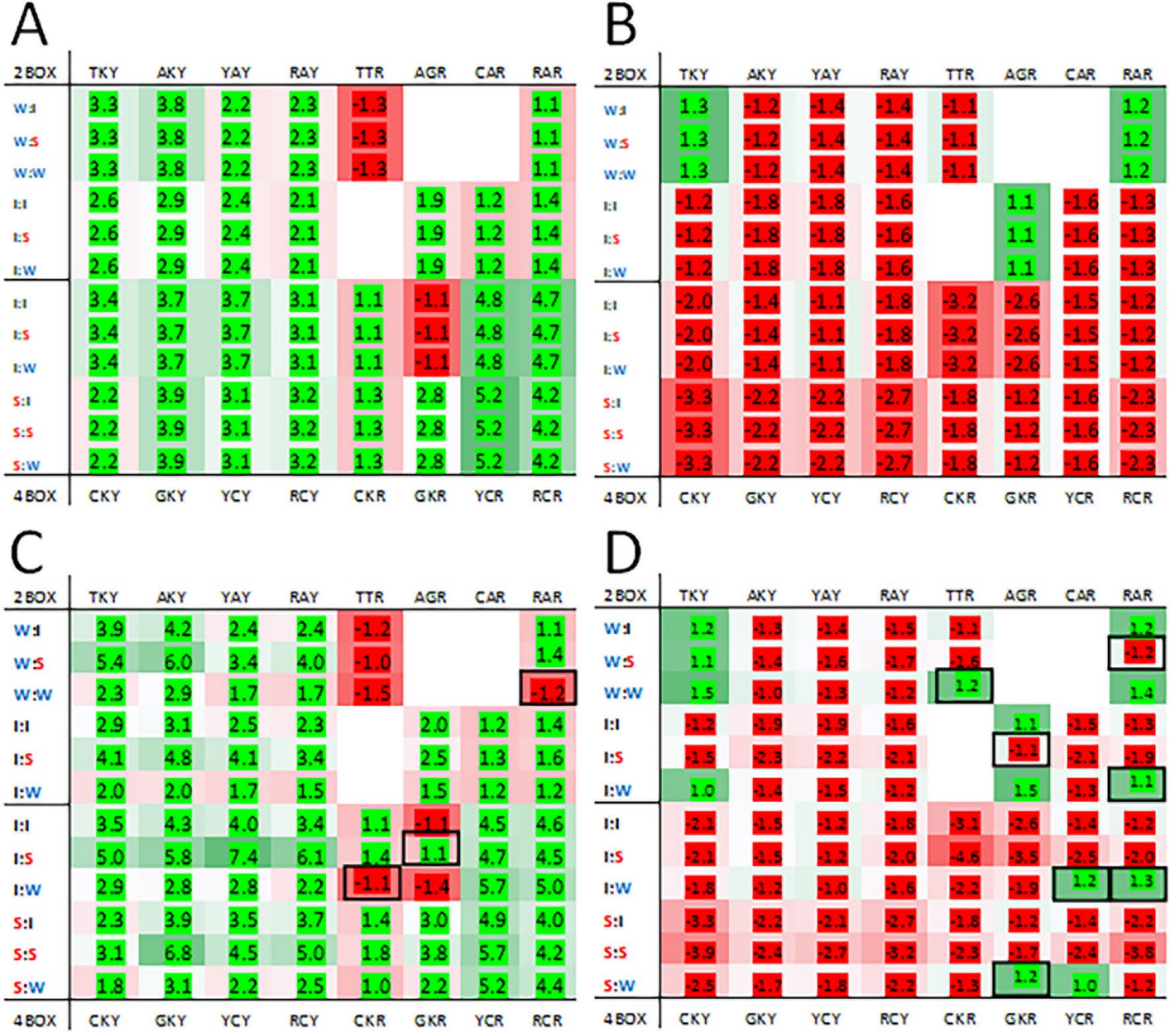
Heatmaps for Coronaviridae (**A** and **C**) and Herpesviridae (**B** and **D**). Heatmaps from the expected codon pairs are shown in the top panels (**A** and **B**) and heatmaps from the observed codon pairs in the lower panels (**C** and **D**). Features of the heatmaps are described in the “Methods” section. Black boxes indicate cells with biases opposite of the expected and with a frequency greater than 1.05 for either observed or expected ratio.

The difference in expected bias and observed bias at the third position of codon pairs is not the same for each virus family but some trends were observed. To illustrate this, heatmaps for Coronaviridae (positive-strand RNA viruses) and Herpesviridae (double-stranded DNA viruses) are provided (Fig. 3). For the AU-rich Coronaviridae genomes the expected bias for the 1st codon ending in a pyrimidine is for the third nucleotide to be a thymine regardless of the network interaction characteristics of the 2nd codon. This is illustrated with the left side of the heatmap being entirely green (Fig. 3, panel A). For 1st codons ending in a purine the bias is for adenosine (green on the right side of the heatmap) except for the TTR and GTR codon groups. The purine bias obtained using the observed data differs from the expected biases. There is an A3 purine bias for GTR for the I:S codon pairs and a G3 bias for CTR and AAR codons in I:W and W:W codon pairs respectively (Fig. 3, panel C). In the GC-rich Herpesviridae genomes some of the observed biases (Fig. 3, panel D) also differ from the expected bias (Fig. 3, panel C). The observed bias at the third position favors thymine for TGY for I:W codon pairs. Adenosine is favored at the third position for: TTR for W:W pairs; TCR and ACR for I:W pairs; and GGR and CCR for S:W pairs. These Herpesviridae biases are all specific to codon pairs with the 2nd codon having ‘weak’ network interaction characteristics. The purine biases that favor G (AGG and AAG) are both for codon pairs with the 2nd codon having ‘strong’ network interaction characteristics (I:S and W:S codon pairs respectively). For both the Coronaviridae and the Herpesviridae the observed biases that trend toward the third nucleotide bias exemplified by the rest of the genome (A and T for Coronaviridae, and G and C for Herpesviridae) are for codons paired with a ‘strong’ 2nd codon. The observed biases that trend away from dominant genome biases are for codon pairs with a ‘weak’ 2nd codon.

**Figure 4:**
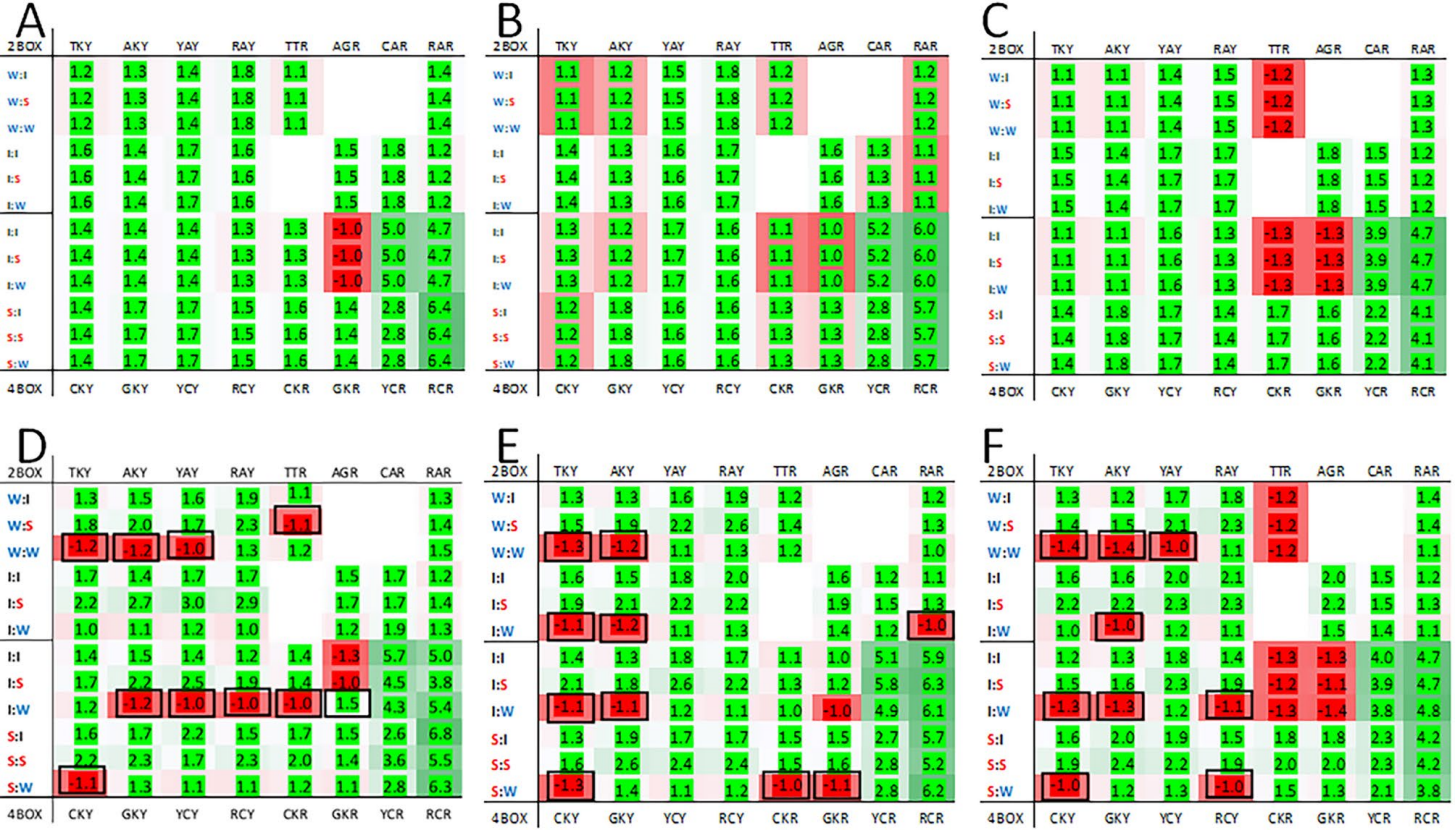
Heatmaps for Filoviridae (**A** and **D**), Paramyxoviridae (**B** and **E**), and Rhabdoviridae (**C** and **F**). Heatmaps for the expected X3 bias for codon pairs are shown in the top panels and heatmaps for the observed X3 bias for codon pairs are shown in the lower panels. Features of the heatmaps are described in the “Methods” section. Black boxes indicate cells with biases opposite of the expected and with a frequency greater than 1.05 for either observed or expected ratio.

Coronaviridae and Herpesviridae are distinct families of virus belonging to different taxonomic kingdoms (Orthornavirae and Heunggongvirae respectively)^20^. To confirm that the trend in the direction of the biases in these diverse AU- or GC-rich families was not coincidental we looked for patterns in other viral families. Filoviriadae, Paramyxoviridae and Rhabdoviridae are all negative strand RNA viruses belonging to the same kingdom (Orthornavirae), phylum (Negaviricota), class (Monjiviricetes) and order (Mononegavirales)^20^. All have AU-rich genomes and this is reflected in the bias for T3 and A3 in the heatmaps generated using expected codon frequencies (Fig. 4, upper panels). Each of these viral families have biases for cytosine or guanosine at the third position when the 2nd codon has ‘weak’ network interaction (Fig. 4, lower panels). This trend away from the prevailing expected genomic bias is similar to the trends described for Coronaviridae and Herpesviridae above. However, there are differences in the number of codon pairs with these biases and there are additional biases unique to some viral families (TTA for W:S and CGG for I:W in Filoviridae; and GAG for I:W in Paramyxoviridae). This suggests that there is some similarity in third position biases when network interactions of the 2nd codon are considered but these biases are not ubiquitous among related virus families.

Codon-pair data is expected to correlate with phylogeny^5,6^. We compared the number of cells in the heatmaps that differed in third nucleotide bias (color, not magnitude) between the various virus families (Table 1). There were 10–15 differences between the Mononegavirales family members (Filoviridae, Paramyxoviridae and Rhabdoviridae). The range of differences increases to 10–24 when the other Negaviricota phylum members Bunyavirales and Orthomyxoviridae were included. The greatest number of differences was observed when the RNA viruses were compared to Herpesviridae which have DNA genomes (Table 1). A Kruskal–Wallis test was performed using the codon pair frequency data. The test showed that there was a statistically significant difference (p < 0.01) for 3 of the heatmap biases among the Mononegavirales family members. There were 36 biases with statistically significant differences when the Negaviricota phylum members were included and 54 statistically different biases when Herpesviridae was included. These data support the idea that codon-pair patterns can be used to identify phylogenetically related organisms^5,6^. A similar analysis of the number of different cells in the heatmaps of some DNA viruses (Supplementary Fig. 4) was performed. The results from this agreed with the recent placement of the single-stranded DNA virus family Parvoviridae with the double-stranded DNA virus families Polyomaviridae and Papilliomaviridae^20^ (Supplementary Table 1). This result was supported by the Kruskal–Wallis testing of the codon pair frequency data with only 18 significantly different heatmap biases identified among the Parvoviridae, Polyomaviridae and Papilliomaviridae. Inclusion of Adenoviridae or Poxviridae resulted in 24 or 39 significantly different heatmap biases respectively.

**Table 1:** Comparison of viral heatmaps. The number of differences in third nucleotide bias (color, not magnitude) between heatmaps for the genomes of various viral families are shown. Virus family names in the top row are truncated with ~ replacing viridae.

Genome	Filo ~	Paramyxo ~	Rhabdo ~	Bunya ~	Ortho ~	Corona ~
Paramyxoviridae	15					
Rhabdoviridae	10	14				
Bunyavirales	11	10	14			
Orthomyxoviridae	19	23	12	24		
Coronaviridae	11	16	13	9	25	
Herpesviridae	65	69	59	71	49	70

**Figure 5:**
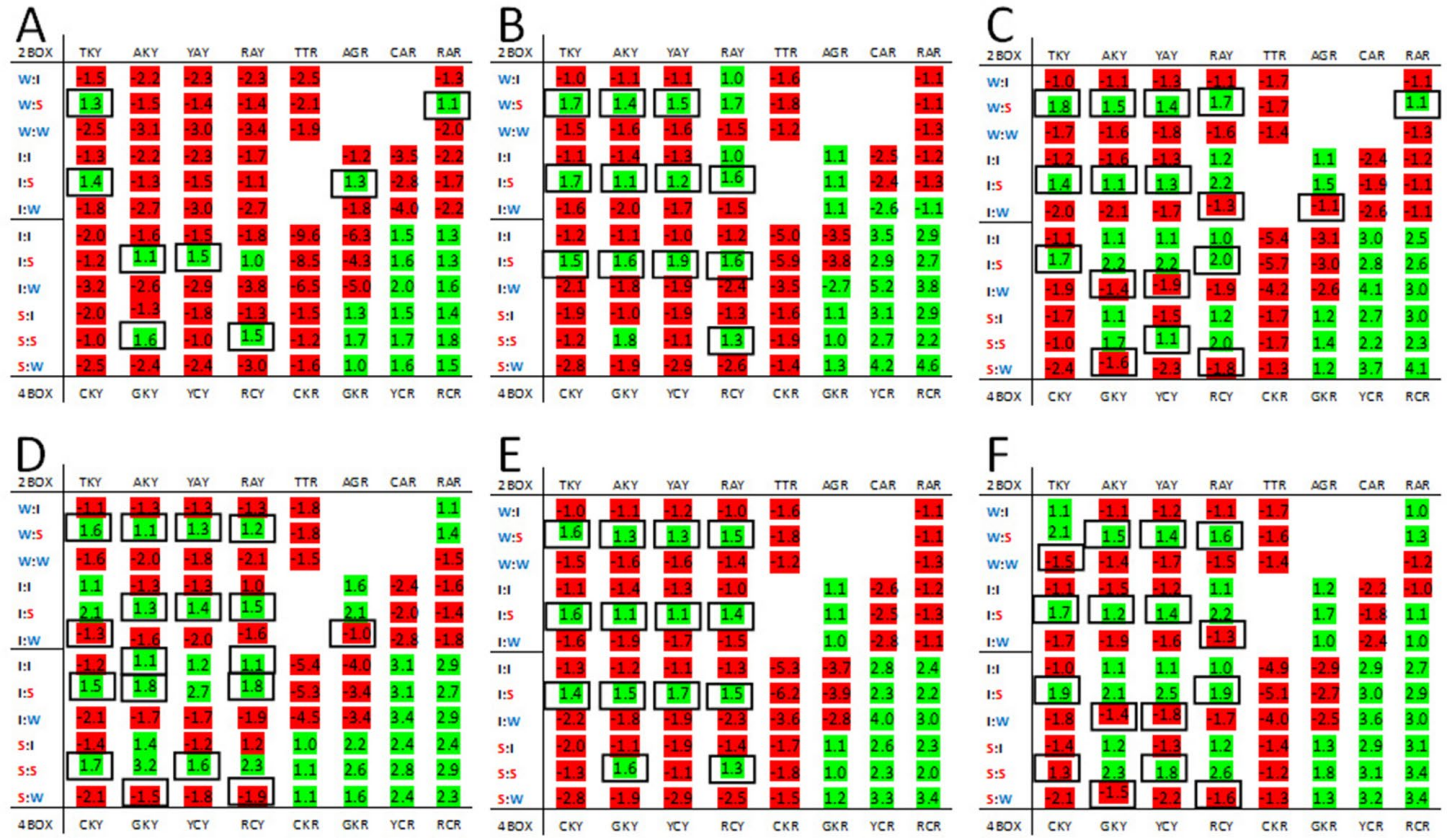
Heatmaps for *Takifugu rubripes* (pufferfish) and *Danio rerio* (zebrafish) (**A** and **D**), *Homo sapiens* (human) and *Myotis lucifugus* (brown bat) (**B** and **E**), and *Gallus gallus* (chicken) and *Columba livia* (pigeon) (**C** and **F**). Features of the heatmaps are described in the “Methods” section. Black boxes indicate cells with biases opposite of the expected and with a frequency greater than 1.05 for either observed or expected ratio.

We compared the heatmaps from humans and some model organisms to examine the patterns in genomes from cellular organisms. The heatmaps from a variety of organisms were assessed but here we only present data from select vertebrates (listed in Supplementary Table 2). Two heatmaps are provided for fish (*Takifugu ruripes* and *Danio rerio*), mammals (*Homo sapiens* and *Myotis lucifugus*), and birds (*Gallus gallus* and *Columba livia*). Patterns were observed in third position bias in the vertebrate heatmaps (Fig. 5). Among all vertebrate genomes analyzed there is a trend for third nucleotide to be cytosine in the 1st codons ending in a pyrimidine (a predominantly red trend on the left side of the heatmaps in Supplementary Fig. 5 and Fig. 5). There is a notable exception to this trend in heatmaps generated using observed codon-pair data: a bias for thymine is observed when the 2nd codon in the pair has ‘strong’ network interaction characteristics. This differs from the virus examples given above which have biases against the predominant trend when the 2nd codon has ‘weak’ network interaction characteristics. The expected pyrimidine bias is not absolute. There is a bias for thymine for: the GTY codon group for zebrafish, human, chicken and pigeon; the TCY codon group for zebrafish, chicken and pigeon; the AAY codon group for human; and the TGY codon group for zebrafish (Supplementary Fig. 5). When these groups are paired with 2nd codons with ‘weak’ network interaction characteristics there is a bias for cytosine (panels B, C, D and F in Fig. 5).

There are fewer differences between the expected and observed biases for 1st codons ending in a purine. In some instances, the bias for a particular third nucleotide is maintained in both the expected and observed heatmaps. For example, the bias for the GCR, YCR and RCR groups is for adenosine and the bias for the TTR, CTR, GTR and CAR groups is for guanosine (Supplementary Fig. 5 and Fig. 5). For the remaining 1st codon groups ending in a purine (CGR, AGR and RAR) the expected bias differs among the species (Supplementary Fig. 5). Where the observed data differs from the expected the purine bias is for adenosine when 2nd is ‘strong’ and guanosine when 2nd is ‘weak’ (panels A, C and D in Fig. 5). However, most differences between the expected and observed third position biases in vertebrates tended to be in 1st codons ending in a pyrimidine (left side of heatmaps) when the 2nd codon had ‘strong’ network interaction characteristics.

We compared the number of cells in the heatmaps that differed in third nucleotide bias between the various vertebrate families (Table 2). The vertebrates are all in the same phylum and the number of differences ranged from 4 to 19. The mammalian heatmaps were most similar to each other (4–9 different codon-pair group biases) and the two fish the most different (19 different codon-pair group biases). The greatest number of differences (19) were observed between pigeon and fugu heatmaps, and pig and zebrafish heatmaps. A Kruskal–Wallis test using the codon pair frequency data showed that there was a statistically significant difference (p < 0.01) for two of the heatmap biases among the Chordata phylum members. The range of differences between the Chordata phylum heatmaps (4–19, Table 2) is similar to the range of differences for viruses in the Negaviricota phylum (10–24, Table 1).

**Table 2:** Comparison of vertebrate heatmaps. The number of differences in third nucleotide bias (color, not magnitude) between heatmaps for the genomes of various vertebrates are shown.

Genome	Human	Pig	Bat	Chicken	Pigeon	Fugu
Pig	9					
Bat	4	7				
Chicken	6	14	9			
Pigeon	12	17	9	7		
Fugu	11	9	12	17	19	
Zebrafish	15	19	10	6	7	19

**Figure 6:**
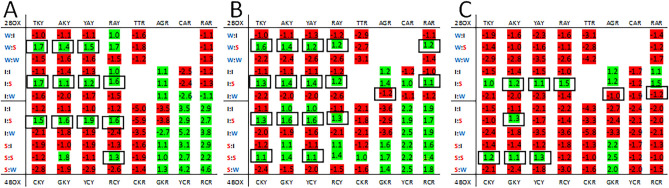
Heatmaps for *Homo sapiens* (human) (**A**), Flaviviridae (**B**), and Culicidae (mosquito) (**C**). Features of the heatmaps are described in the “Methods” section. Black boxes indicate cells with biases opposite of the expected and with a frequency greater than 1.05 for either observed or expected ratio.

For a comparison between host and viral genomes we selected positive-strand RNA viruses from the Flaviviridae family. There are several flaviviruses known to infect both humans and insects but there are also insect specific flaviviruses^21^. This enables the comparison of codon-pair features of virus with two divergent host genomes, and comparison of viral genomes within the same family that have a more restricted host range^4^. First, we compared the heatmaps generated using human, mosquito and Flaviviridae observed data (Fig. 6). There are several examples in all three heatmaps of observed third position biases that differ from the expected bias for 1st codons ending with pyrimidines (the I:S codon groups AGY, CAY, GAY and GTY). There are additional biases shared between human and Flaviviridae heatmaps (compare panels A and B in Fig. 6: The W:S codon groups TTY, ATY and TAY; and I:S codon groups TGY, CTY, CAY and ACY), and between the mosquito and Flaviviridae heatmaps (compare panels B and C in Fig. 6: The S:S codon groups CGY, and CCY; and I:S codon groups AGR). In sum, for observed biases that differ from the expected bias, 11/17 observed Flaviviridae biases imitate the 12 observed human biases, and 7/17 imitate the 9 observed mosquito biases.

**Figure 7:**
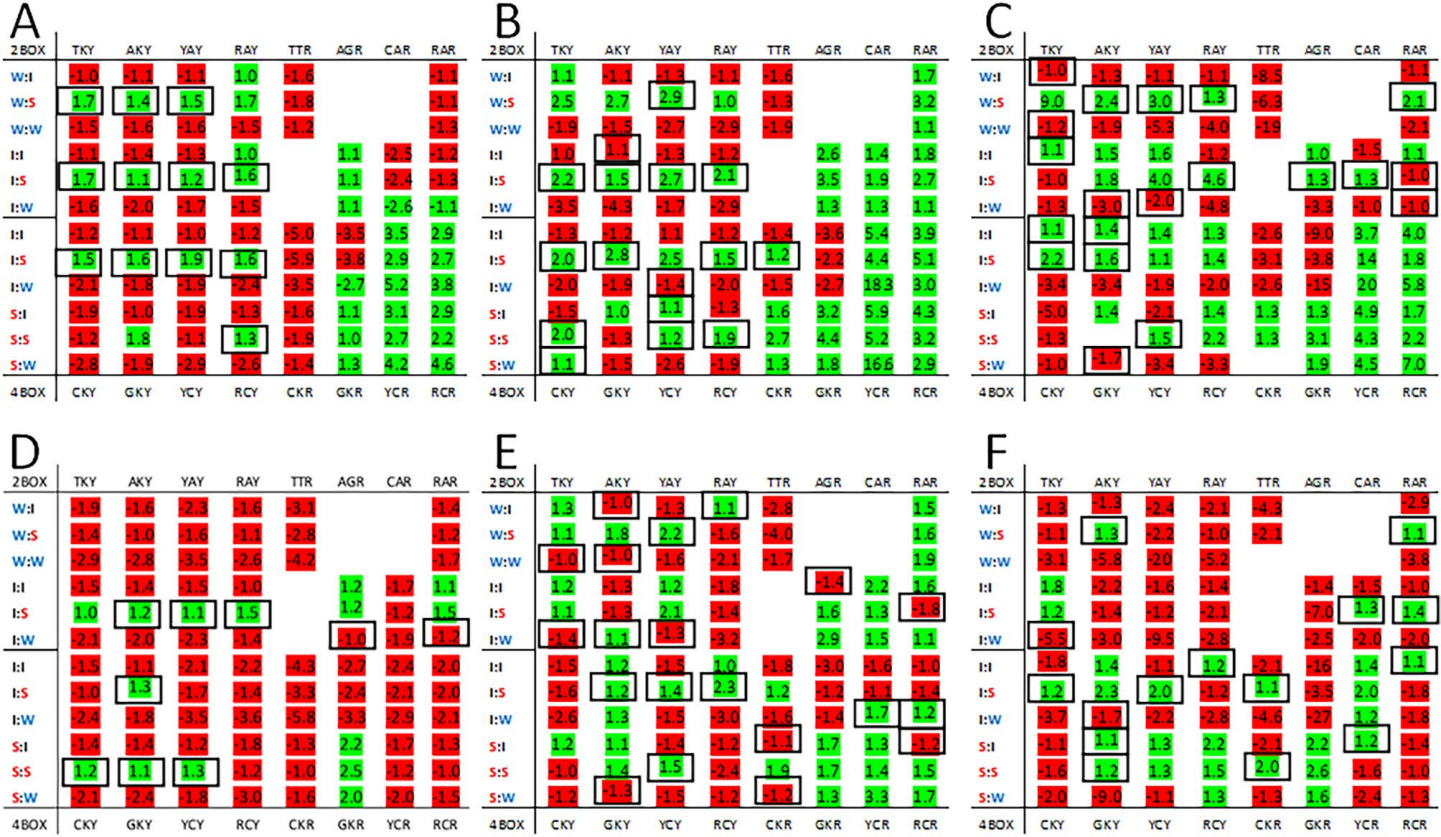
Heatmaps for *Homo sapiens* (human) and *Culicidae* (mosquito) (**A** and **D**), Dengue and Yellow fever viruses (**B** and **C**), and insect specific Aedes and Culex flaviviruses (**E** and **F**). Features of the heatmaps are described in the “Methods” section. Black boxes indicate cells with biases opposite of the expected and with a frequency greater than 1.05 for either observed or expected ratio.

To investigate whether the observed biases correlated with specific host biases we analyzed two insect-specific flaviviruses and two that utilize two host species (Fig. 7). First, we assessed the variation amongst the flavivirus genomes using heatmaps. We compared the number of cells that had a different bias between each of the flaviviruses and the Flaviviridae family (see Table 3). The smallest number of differences between two genomes was between the Dengue and Yellow fever viruses. These two flaviviruses also had the least number of differences from the Flaviviridae family heatmap. This possibly reflects the data used to compile the Flaviviridae codon and codon-pair usage, which may be comprised of more human disease-centric genomic data and less insect specific flavivirus data. There are fewer codon pairs used to generated data for individual viruses than for a family of viruses and this may lead to less resolution and more skewed values. For example, the bias ratios were greater than 15 for several codon pairs containing a weak 2nd codon (YCR biases for adenosine for Dengue and Yellow fever flaviviruses, and GTR biases for guanosine in Yellow fever and Culex flaviviruses). The Kruskal–Wallis tests using the codon pair frequency data from Flaviviridae, and the Aedes and Culex flaviviruses showed that there were statistically significant differences for 10 of the heatmap biases whereas there were 19 statistically significant different biases among Flaviviridae, Dengue and Yellow fever viruses. In addition, the pattern of predicted codon box biases generated from the expected frequencies differed between the viruses (Supplementary Fig. 6). These data demonstrate limitations of the heatmaps when comparing the differences between individual viral genomes.

**Table 3:** Comparison of flavivirus heatmaps. The number of differences in third nucleotide bias (color, not magnitude) between heatmaps for specific flavivirus genomes is shown. The two insect specific viruses (Aedes and Culex) and two viruses that cause disease in humans (Dengue and Yellow fever) are also compared to the Flaviviridae heatmap.

Genome	Dengue	Yellow fever	Aedes	Culex
Yellow fever	21			
Aedes	26	30		
Culex	33	26	32	
Flaviviridae	17	9	32	23

We compared individual flavivirus third nucleotide biases to the human and mosquito genomes by comparing the number of cells that had a different bias (Table 4). The Dengue and Yellow fever flavivirus had the fewest differences (22 and 19 respectively) from the human heatmap. The Culex flavivirus had the fewest differences (27) from Culicidae. The Kruskal–Wallis analyses identified 28 significantly different heatmap biases among Dengue and Yellow fever flaviviruses and the human genome. There were 29 significantly different heatmap biases among Dengue and Yellow fever flaviviruses and Culicidae. There were fewer differences when the Aedes and Culex flaviviruses were compared to human and Culicidae genomes, 17 and 14 respectively. This suggests that these individual virus genomes are better matched to the respective host types. The Flaviviridae, Coronaviridae, and Herpesviridae heatmaps were also compared to the human and mosquito heatmaps. The number of differences ranged from 13 to 65 with Flaviviridae being the most similar to the human heatmap and Coronaviridae being the most different from mosquito heatmap. When the Kruskal–Wallis analysis of the codon pairs underpinning the heatmaps were assessed, there were more significantly different biases when any two of the viral genera were analyzed with the Culicidae genome than with the human genome. This correlates well with there being more heatmap differences between each of those viruses and Culicidae genome than with the human genome.

**Table 4:** Comparison of flavivirus heatmaps with heatmaps from *Homo sapiens* and Culicidae. The number of differences in third nucleotide bias (color, not magnitude) between heatmaps for specific flavivirus genomes is shown. Heatmaps from three viral families (Flaviviridae, Coronaviridae and Herpesviridae) are also compared to the *Homo sapiens* and Culicidae heatmaps.

Genome	Dengue	Yellow fever	Aedes	Culex	Flavi-viridae	Corona-viridae	Herpes-viridae
*H. sapiens*	22	19	33	32	13	46	35
Culicidae	37	36	37	27	28	65	24

## Discussion

This research shows that there are biases in third nucleotide position of the 1st codon that are associated with the network interaction characteristics of the codon pair. The patterns revealed by these biases correlate well with the phylogeny of viruses and cellular organisms. We observed the same bias at the third nucleotide for some virus and host codon groups even though the expected biases differed among the organisms. The most striking example of this was with the Flaviviridae, mammals and bird biases. When we extended our analysis to flaviviruses with restricted host range and flaviviruses that infected more than one host we observed a better correlation between viruses that caused disease in humans than for viruses that are insect specific (Table 4). A similar result was obtained when viral codon usage was compared to the host tRNA pool for a group of positive-strand RNA viruses^3^. We did not observe obvious correlations between virus and host genomes biases in other virus families we analyzed (*H. sapiens* and Herpesviridae biases for example). In fact, there was no obvious correlation for several virus/host analyses, including viruses with prokaryote and plant hosts (Supplementary Fig. 7). Similar observations have reported by others^14,15^, indicating that the similarity we observed between Flaviviridae and host biases may be the exception rather than the rule. Differences between virus families may relate to the importance of fast protein production and if it is regulated via the availability of the transcripts or post-transcriptionally^16–18^. This may be investigated through more detailed comparisons of ORFs that are translated at different times during viral infection, or by comparison of viruses with short and long infection cycles.

The exponentially increasing size of available sequence data creates computational challenges and this has led to the development of more efficient methods of comparing genomes^22,23^. Our comparison of third position bias in codon pairs adds a new tool for analysis of all types of genomes. We identified similar patterns in phylogenetically related organisms from consolidated codon-pair frequency data. Our analyses using data from several virus families demonstrate that the differences in heatmaps can be used to infer phylogenetic grouping of viruses (Tables 2 and 4, and Supplementary Table 1). An earlier virus classification system, the Baltimore system^24^, placed Parvoviridae (with a single-stranded DNA genome) in a different class from Polyomarviridae and Papillomaviridae (which have double-stranded DNA genomes). Recently these three families were grouped in the same Kingdom and Phylum, as these double-stranded DNA viruses appeared to evolve from the single-stranded viruses^20,25^. The small number of differences between the heatmaps of these viral families compared to other double-stranded DNA virus families, Adenoviridae and Poxviridae, (Supplementary Fig. 4 and Supplementary Table 1), agrees with the ICTV phylogeny.

The base composition of groups of phylogenetically related viruses are similar but may differ from that of the host, especially for viruses that infect eukaryotes^14^. Also, the frequency and type of mutational error viral polymerases introduce also differs among viruses^26^. Dinucleotide composition contributes to codon-pair bias in some viral genomes^1,15^. And the depletion of CpG which has been shown to result in viral attenuation^13,16,27^. However, the extent of CpG depletion in viral genomes and impact on viral viability may depend on the viral host species^17,18,28^. From our heatmaps we would expect to see CpG depletion as a pyrimidine preference for C over T at the third codon position of the 1st codon when the second codon is ‘strong’ or a purine preference for A over G at the third nucleotide of CCR (alanine), GGR (proline), ACR (threonine), and TCR (serine) codons. Most of the trends we describe below did not appear to be associated with CpG depletion.

We investigated the hypothesis that biases in the third position of viral codon pairs would be similar to host biases because they use the same protein production machinery. Alignment of RNA-dependent RNA polymerase amino-acid sequences lead to the clustering of plant and animal viruses in dendrograms^29^. The common feature in that analysis was that the clustered viruses either infected insects or used insects as vectors. When we investigated whether viruses from the same genera had biases that converged with specific host biases, we observed fewer differences between *H. sapiens* and the Dengue and Yellow fever flavivirus biases than between *H. sapiens* and Culicidae flavivirus biases (Table 4). The reciprocal observation was made when the virus biases were compared to Culicidae biases. This suggests that codon-pair biases might also evolve to better match host biases. However, our analyses were limited one family of positive-stranded RNA viruses preventing such a general conclusion to be reached. Indeed, we detected many instances where viral biases did not match host genome biases. This is perhaps not surprising given the diversity of viral genomes that can infect one species (Coronaviridae and Herpesviridae in Fig. 3 for example). In addition to the disparate virus and host biases described here we also found differences between viruses and their hosts from yeast, plant and bacterial genera (Supplementary Fig. 7). This suggests that the third position biases in codon pairs correlate more with viral genera and are not strictly a host adaptation.

A comparison of a variety of genomes revealed some trends. When the observed third nucleotide biases differed from the expected bias, it was more often associated with codon pairs in which the 2nd codon had either ‘strong’ or ‘weak’ network interactions and was rarely associated with ‘intermediate’ codons. For pyrimidine bias we report a C3 preference (away from the dominant T3 pyrimidine bias) for codon pairs with a 2nd codon with ‘weak’ network interactions for the Filoviridae, Paramyxoviridae, Rhabdoviridae and Orthomyxoviridae families (Fig. 4 and Supplementary Fig. 3). The bias for T3 (away from the dominant C3 pyrimidine bias) was present in Flaviviridae and vertebrate genomes (Figs. 5 and 6) for some codon pairs where the 2nd codon had ‘strong’ network interactions. Both of these trends were observed in the Parvoviridae, Polyomaviridae and Papillomaviridae families which have a more varied mix of expected pyrimidine biases at the third nucleotide (Supplementary Fig. 4). These trends lead to specific dinucleotides (CA, AT, TC and TG) spanning the codon pair. Our heatmaps don’t distinguish between codons with ‘intermediate’ strength network characteristics that begin with a purine or a pyrimidine so we cannot readily determine if these trends are the result of dinucleotide bias at the nucleotides spanning the codon pair or are solely due to network interaction strength.

The biases involving purines at the third nucleotide position present a different picture. Most of the virus genomes analyzed had a mix of expected biases with neither A3 nor G3 dominating the right side of the heatmaps. The observed A3 bias for codons with expected G3 were with adjacent ‘strong’ 2nd codons and observed G3 bias for codons with expected A3 were with adjacent ‘weak’ codons for several organisms described (T*. rubripes*, *D. rerio* and *G. gallus*, Fig. 5; and Flaviviridae and Culicidae, Fig. 6). These trends lead to AG, AC, GA and GT dinucleotide pairs spanning the codon pair. But biases for several genomes did not follow this pattern: Herpesviridae (Fig. 3); and Parvoviridae, Polyomaviridae, Papillomaviridae, Adenoviridae and Poxviridae families, (Supplementary Fig. 4). For these viruses the observed G3 biases that differed from the expected A3 had adjacent ‘strong’ codons and the observed A3 biases that differed from the expected G3 often had adjacent ‘weak codons. These trends lead to GG, GC, AA and AT dinucleotides spanning the codon pair. These trends indicate that the bias in the third position (in a codon pair) correlates with the phylogenetic grouping and affects the dinucleotide pair frequency spanning codon pairs. The similarities in trends among diverse organisms suggests that the biases may be important for protein translation.

There is additional information that can be gleaned from the heatmaps that was not explored: the magnitude of a ratio indicating the bias and statistical modeling. An ad hoc inspection of the heatmaps suggests that some of the biases observed for one virus were paralleled by differences in the magnitude of expected and observed frequencies for closely related viruses even if a change in Y3 or R3 bias didn’t occur. The magnitude of the expected bias was similar for ‘intermediate’, ‘strong’ or ‘weak’ 2nd codons, but the magnitude of the observed bias varied. We were not able to identify a statistical model applicable to the heatmap itself but were able to apply a statistical analysis of the codon-pair frequencies underlying the biases described in the heatmaps. Here we show the heatmaps and Kruskal–Wallis testing can facilitate comparison of genomes with limited codon-pair data.

The significance of the biases described here has not been established. Because the difference is associated with network interaction characteristics, we hypothesized that the magnitude of the bias is linked to protein production. Protein production is an integral part of the virus lifecycle. The use of synonymous codons can lead to different protein folding and reduced cell fitness^30,31^. It has also been shown that codon pairs, rather than the individual codons themselves, are able to affect the rate of translation^32^. An analysis of codon-pair frequencies in proteins of high and low abundance identified codon pairs that were preferred in a range of organisms including *C. elegans*, *D. melanogaster*, and *S. cerevisiae*^5^. The high protein abundance sequences had more preferred codon pairs compared to the low protein abundance sequences (40 versus 16 respectively) but both sets were dominated by codon pairs that included ‘weak’ network interaction characteristics. This suggests that third position bias in the context of network interactions is not tightly correlated with protein abundance. However, it has been demonstrated that the anticodon:codon interaction at the third position affects translation speed^33^. It is likely that the certain codon pairs alter the speed of translation and the folding of the nascent protein is affected^34^.

The tRNA pools available for protein production are not static and tRNA abundance can alter protein synthesis^35^. Stress and interferon alter the activity of enzymes that aminoacylate or modify tRNAs^36,37^. Defects in tRNA modification, particularly with the wobble nucleotide in the anticodon loop (tRNA position 34) which pairs up with the third nucleotide of a codon, can alter the tRNA pool available to decode certain nucleotides. An mRNA containing codons that depend on tRNA modifications may be translated at lower rates or misfolded. Fewer proteins or misfolded proteins could disrupt the protein homeostasis affecting cell phenotypes and viability^34^. It has been shown that the codon usage for the influenza PB1 gene better matches the interferon-induced tRNA pool^38^. It would be interesting to determine if there is a correlation between third nucleotide bias in ORFs from other viral genomes and the modification status of the host tRNA population. Such a correlation could be identified in using our heatmaps by comparing ORFs from commonly expressed host proteins, ORFs of proteins upregulated during stress conditions and viral ORFs.

Buchan et al., 2006, analyzed over- and under-represented codon pairs in a variety of organisms^9^. They observed that the observed frequency of the third position in the first codon and the first position in the second codon differed significantly from what was expected. This leads to differences in the observed codon-pair frequencies and dinucleotide patterns. Additionally, Buchan et al., identified correlations between the third position in the first codon and the second or third positions of the second codon. Similarly, we have identified patterns that are derived from the observed correlations between the third position of the first codon and the first two positions of the second codon. Buchan et al., observed that prokaryote genomes generally had more than expected rare codon pairs and eukaryote genomes generally had fewer than expected rare codon pairs. We observed that, in general, the observed biases tended to occur when the second codon had strong or weak network interactions in both cellular and viral genomes. Either of these observations could be linked to other genomic features such as GC content. This remains an area open to future research.

In conclusion, we have described a simple method for generating heatmaps that allows one to view third position biases in the 1st codon of the codon pairs in a genome. Classifying codons according to their network interactions strength combines frequencies from similar codons and reduces the number of datapoints for comparison from several thousand to less than one hundred. Identification of these biases has several applications including exploring changes in viral genomes as they adapt to new hosts, supplementing taxonomic classification, developing attenuated viruses for vaccines, and improving the expression of proteins in foreign hosts.

## Methods

Codon usage tables (CUTs) and codon-pair frequency data were retrieved from the HIVE CoCoPUTs database (https://hive.biochemistry.gwu.edu/cuts/about accessed August 4, 2021)^19^. TAXIDs represent nodes in taxonomic trees and features of the taxon from the branch of the tree can be retrieved from the database at https://www.ncbi.nlm.nih.gov/taxonomy .^39^ TAXIDs were used to retrieve codon and codon-pair data for specific viruses, species or genera. An excel file was created to calculate the expected codon-pair frequencies from the CUTs (see Supplementary File 1). Outputs including codon-pair distribution, frequency of codon pairs with different network interaction strength, and heatmaps indicating bias at the third position are also generated. The network strength characteristics of each codon is as described in Grosjean and Westhof^10^. Briefly, codons with the first two nucleotide positions comprised of G and/or C are designated ‘strong’. Codons with the first two positions comprised of A and/or T are designated as ‘weak’, and the others are designated ‘intermediate’. Codons are described as 4-box codons when four synonymous codons, differing only in the third nucleotide position, exist. Codons for which two synonymous codons exist are described 2-box codons. All the ‘strong’ codons are 4-box codons, and all the ‘weak’ codons are 2-box (using ATT and ATC for isoleucine but not ATA; see Supplementary Fig. 1).

The heatmap indicates the bias at the third nucleotide position of the 1st codon of a codon-pair where both the 1st and 2nd codons are grouped according to network interaction characteristics (Fig. 1). Only the bias between the two possible purines (A or G), or the two possible pyrimidines (T or C) is evaluated. Green shading indicates the thymine or adenosine is the preferred nucleotide and red shading indicates cystine or guanosine is the preferred nucleotide. The rows of the heatmaps are aligned to facilitate comparison of the third position bias among the 2-box codon groups, and among the 4-box groups according to the strength of the network interactions of the 1st codon (Fig. 1). The rows are further delineated to allow comparison of groups with a ‘weak’, ‘intermediate’ or ‘strong’ 2nd codon. The columns of the heatmap are arranged so that groups in which the 1st codon ends in a pyrimidine are on the left and groups with the 1st codon ending in a purine are on the right. Termination codons and those for tryptophan, methionine and the ATA isoleucine codon are not included in the heatmaps. The bias in the heatmaps is shown as the ratio of frequencies for –(T/C) and –(A/G) codons. Ratios greater than 1 are shown in green. Where C and G were more frequent at the third nucleotide than T and A respectively the reciprocal ratio is shown in red (Fig. 1). The green/red shading of cells was generated using Microsoft Excel (see supplementary file 1). This shading was based on the range of numbers in the table and, for some genomes (like those with high or low GC content or highly skewed codon usage), both green and red shading could be associated with cells that were the same sign (positive or negative). Although this shading may be useful for visualizing the magnitude of the biases it is not always useful for visualizing whether trends are positive or negative. All tables were transferred to a Microsoft Word document and positive ratios were manually highlighted green and negative ratios highlighted red to provide clarity for the figures in this work. On the left side of the heatmap the network interaction characteristics of the codon pairs for each row is indicated (W:I, weak-intermediate; W:S, weak-strong; W:W, weak-weak; I:I, intermediate-intermediate; I:S, intermediate-strong; I:W, intermediate-weak; S:I, strong-intermediate; S:S, strong-strong; and S:W, strong–weak). Above and below the heatmap the 1st codon sequences are shown (see Figs. 3, 4, 5, 6, 7 for example). Black boxes around bias values indicate cells with observed biases opposite of the expected and with a frequency greater than 1.05 for either the observed or expected ratio.

The Kruskal–Wallis statistical test was performed for our heatmap biases among genomes. Briefly, the frequencies of codon pairs for genera listed in Tables 1 through 4 were organized according to the corresponding cells in the heatmaps (Supplementary File 1). The underlying codon pairs for each cell for at least three groups were transferred to a new file (Supplementary File 2) and the rank order determined. The sum of the rank order values was calculated for each group, then squared and divided by the number of contributing values (not all possible codon pairs are represented in each genome or genera). The Kruskal–Wallis H-value was calculated and the probability of the chi-squared distribution calculated. The test was used to identify if the mean codon-pair frequency for each cell in the heatmap was the same among at least three different groups. For comparisons among the different groups the number of heatmap cells with statistically significant differences (p < 0.01) is reported in the text.

## Supplementary Information

Supplementary Information 1.

Supplementary Information 2.

Supplementary Figures and Tables.
