# Supplementary material for: Bias at the third nucleotide of codon pairs in virus and host genomes
Source: Sci Rep. 2022 Mar 16;12:4522. doi: 10.1038/s41598-022-08570-w (PMC8927144; doi:10.1038/s41598-022-08570-w)
Supplement: Supplementary file 3 — Supplementary Figures and Tables. [file 41598_2022_8570_MOESM3_ESM.pdf]

Supplementary Table 1. Bias at the third nucleotide of codon pairs in virus and host genomes. Plant & Ye, 2022

Family	Herpesviridae	Adenoviridae	Poxviridae	Papilliomaviridae	Polyomaviridae
**Adenoviridae**	21				
**Poxviridae**	63	46			
**Papilliomaviridae**	61	44	16		
**Polyomaviridae**	63	46	16	6	
**Parvoviridae**	54	39	22	20	22

Supplementary Table 1. The number of cells in heatmaps that differ in wobble position bias. The heatmaps for each virus displayed in Supplementary Figure 4 were compared and the differences (in the direction of bias, not magnitude) are tabulated.

Species	Kingdom	Phylum	Class	Order	Family
H. sapiens	Animalia	Chordata	Mammalia	Primates	Hominidae
S. scrofa	Animalia	Chordata	Mammalia	Artiodactylia	Suidae
M. musculus	Animalia	Chordata	Mammalia	Rodentia	Muridae
M. lucifugus	Animalia	Chordata	Mammalia	Chiroptera	Vestpertilionidae
T. rubripes	Animalia	Chordata	Actinopterygii	Tetraodontiformes	Tetraodontidae
D. rerio	Animalia	Chordata	Actinopterygii	Cypriniformes	Cyprinidae
G. gallus	Animalia	Chordata	Aves	Galliformes	Phasianidae
C. livia	Animalia	Chordata	Aves	Columbiformes	Columbidae

Supplementary Table 2. The taxonomic classification of vertebrates used for analyses in Figure 5 and Supplementary Figure 5.

Supplementary Figure 1. **Bias at the third nucleotide of codon pairs in virus and host genomes.** **Plant & Ye, 2022**

The codons and associated amino acids are displayed in a circular arrangement with the three nucleotides of each codon arranged from the inner to the outer rings of the circle. The arrangement groups the codons with any combination of guanosine and cytosine in the first two codon positions at the top of the circle. These codons have ‘strong’ network interaction as described in Grosjean and Westhof, 2016; doi: 10.1093/nar/gkw608. Codons with ‘weak’ network interactions are on the bottom of the circle and the codons with ‘intermediate’ network interaction are arranged between the ‘strong’ and ‘weak’.

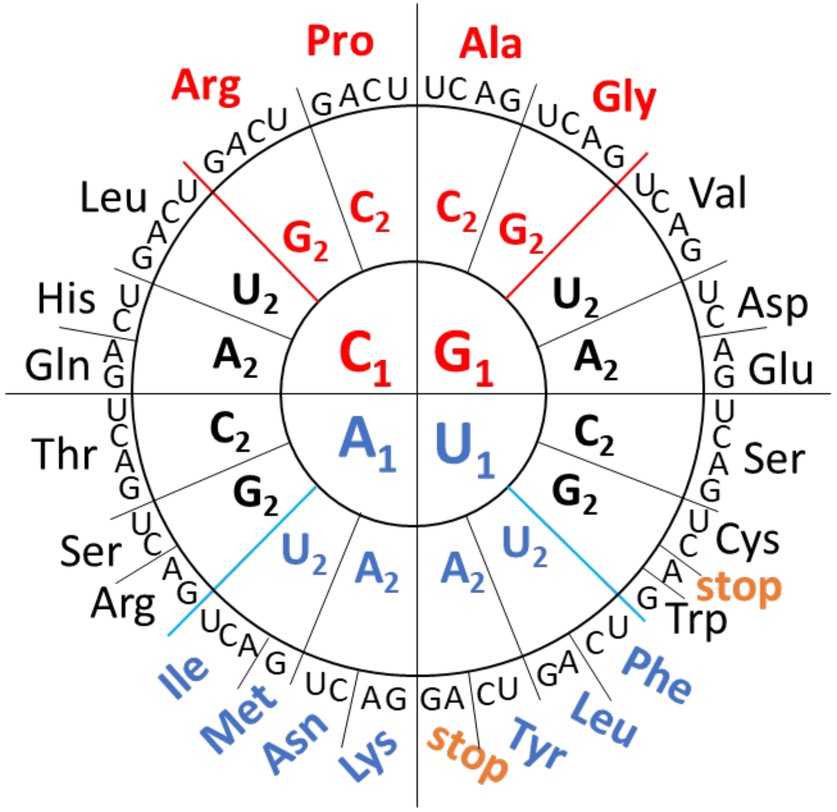

GC at the first two positions of the codon = **Strong**

AU at the first two positions of the codon = **Weak**

Other variations = **Intermediate**

2-box codons end R3 or Y3

Codon-Pairs can be grouped
W:W, W:I, I:I, S:S, S:I, S:W
or with **terminator**

Supplementary Figure 2. Bias at the third nucleotide of codon pairs in virus and host genomes. Plant & Ye, 2022

The distribution of the observed frequencies for codon-pairs from a variety of organisms are shown. The data for each organism were obtained from the CoCoPUTs database, log-transformed using the excel file (Supplementary File 1) and graphed.

Animalia

- Homo sapiens (human)
- Sus scrofa (pig)
- Myotis davidii (bat)
- Gallus gallus (chicken)

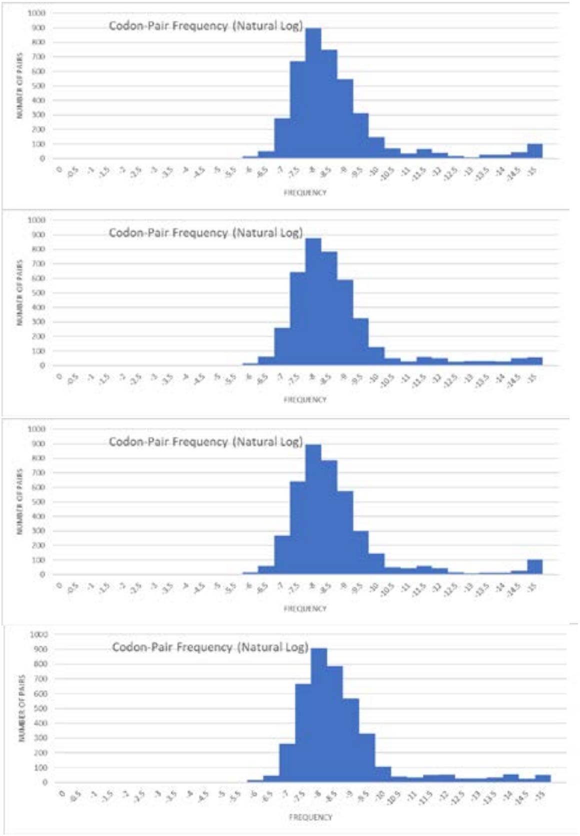

1.8 – 7.8 million codon-pairs

Fungi

- Saccharomyces cerevisiae (yeast)

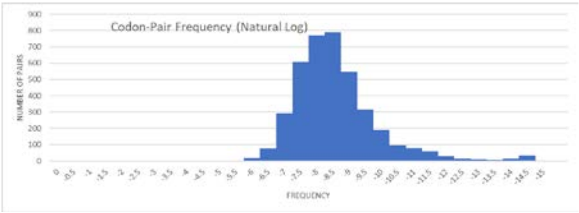

2.9 million codon-pairs

Plantae

- Phoenix dactylifera (palm date, monocotyledon)
- Arabidopsis thaliana (mustard, dicotyledon)

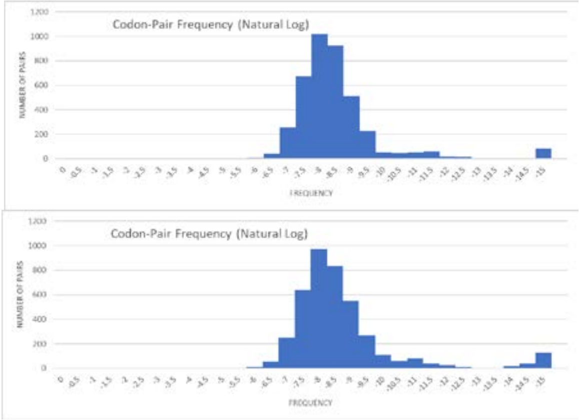

17 - 21 million codon-pairs

Supplementary Figure 2. **Bias at the third nucleotide of codon pairs in virus and host genomes.** **Plant & Ye, 2022**

Gram Negative

- Escherichia coli

Gram Positive

- Bacillus subtillis

Cyanobacterium

- Microcystis aeruginosa

Protobacteria

- Vibrionaceae

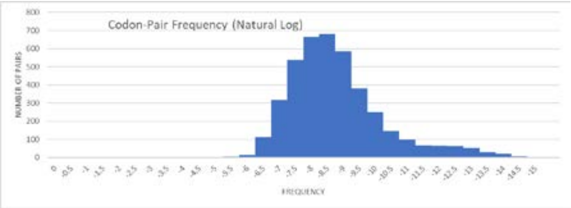

2.8 x 10^{10} codon-pairs

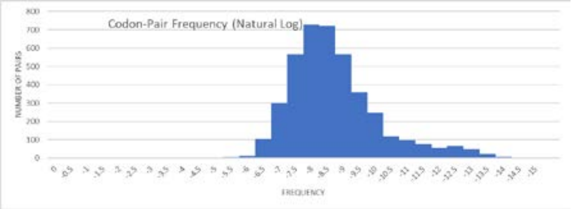

3.7 x 10^{8} codon-pairs

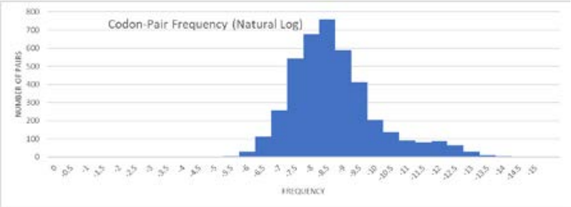

5.2 x 10^{7} codon-pairs

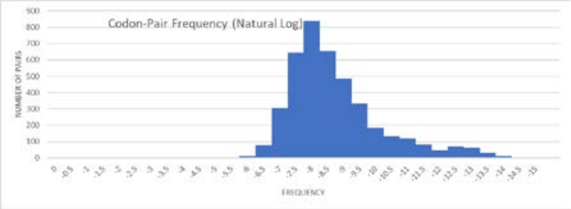

5.9 x 10^{9} codon-pairs

ssRNA-RT Viruses

- VI Retroviridae

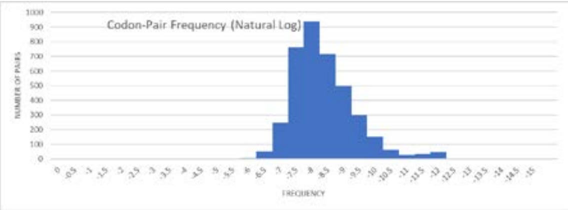

2.1 x10^{5} codon-pairs

dsDNA-RT Viruses

- VII Hepadnaviridae

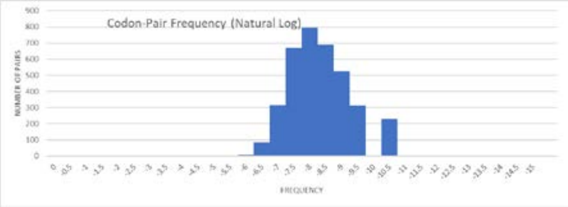

3.7 x10^{4} codon-pairs

Supplementary Figure 2. **Bias at the third nucleotide of codon pairs in virus and host genomes.** **Plant & Ye, 2022**

dsDNA Viruses

- I Herpesviridae
- I Poxviridae
- I Adenoviridae

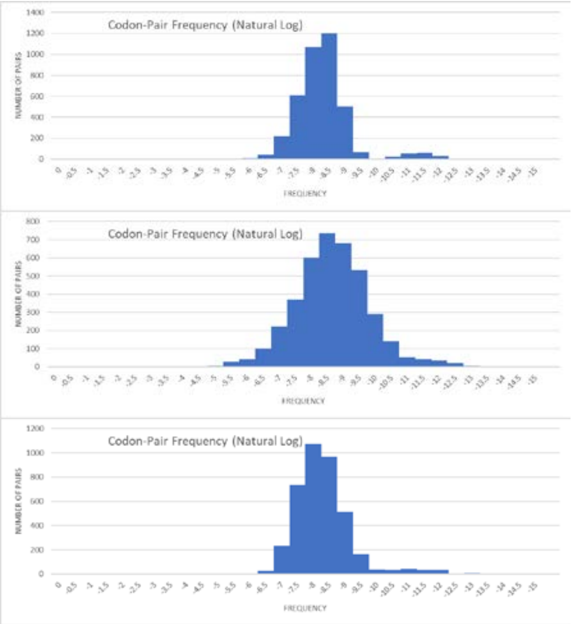

1 – 3.5 million codon-pairs

dsDNA Viruses

- I Papillomaviridae
- I Polymaviridae

dsDNA Viruses

- II Parvoviridae

ssDNA Viruses

- III Reoviridae

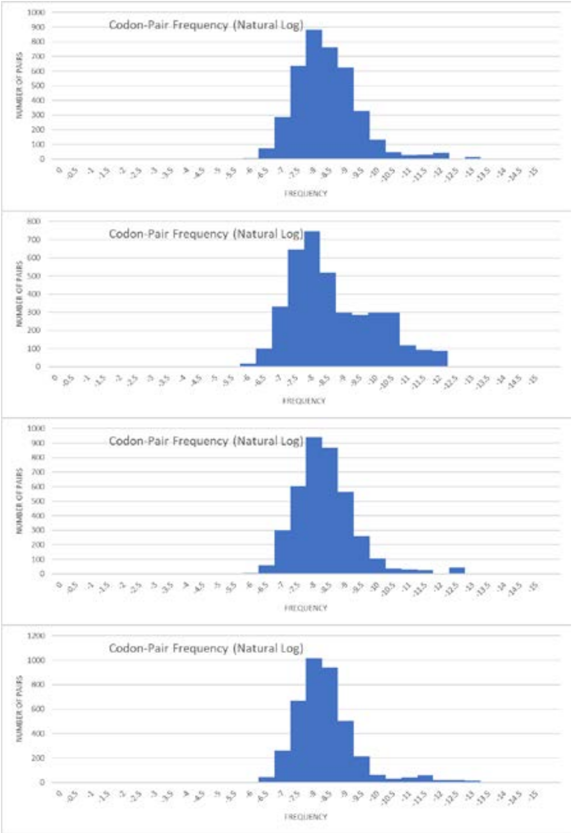

2.3 – 6.4 x10^5 codon-pairs

Supplementary Figure 2. Bias at the third nucleotide of codon pairs in virus and host genomes. Plant & Ye, 2022

(+) ssRNA Viruses

- IV Coronaviridae
- IV Picornaviridae
- IV Flaviviridae
- IV Caliciviridae
- IV Togaviridae

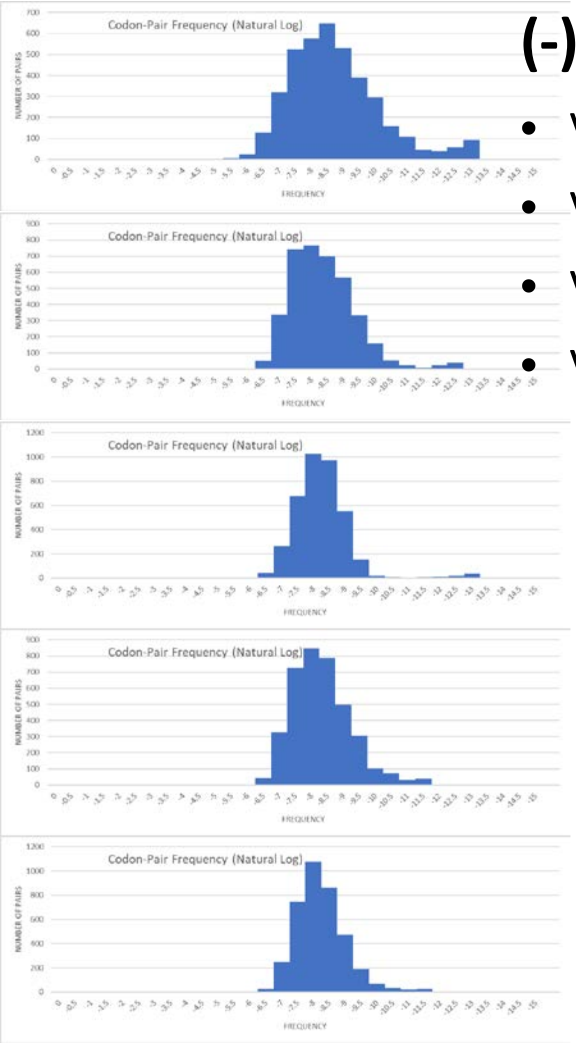

1.5 – 5.8 x10^5 codon-pairs

(-) ssRNA Viruses

- V Paramyxoviridae
- V Rhabdoviridae
- V Orthymyxoviridae
- V Bunyavirales

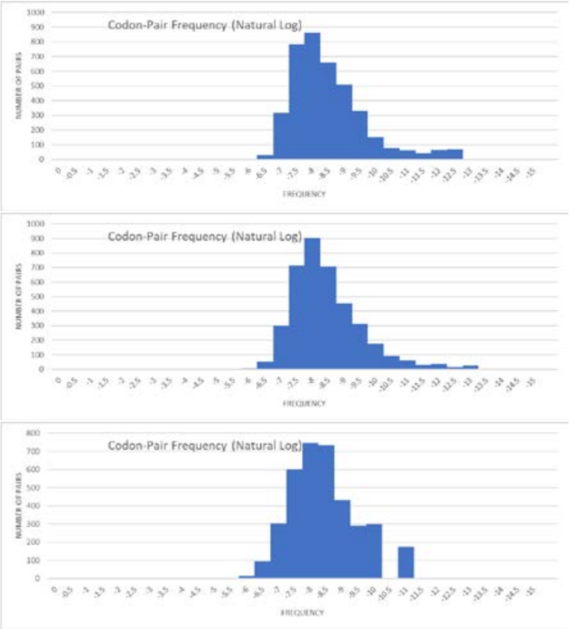

0.7 – 6.7 x10^5 codon-pairs

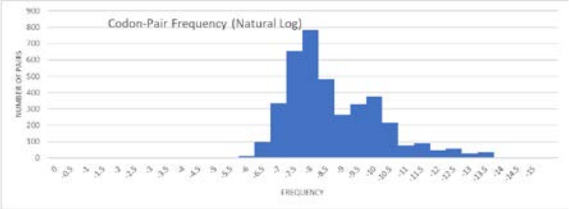

1.1 million codon-pairs

Supplementary Figure 4. Bias at the third nucleotide of codon pairs in virus and host genomes. Plant & Ye, 2022

Heatmaps for viruses belonging to the Cossaviricota phylum in the Shotokuviriae kingdom (**A**, Parvoviridae; **B**, Polyomaviridae; and **C**, Papilliomaviridae), the Peploviricota phylum in the Heunggongvirae kingdom (**D**, Herpesviridae), and the Preplasmiviricota and Nucleocytoviricota phylums in the Bamforviae kingdom (**E**, Adenoviridae; and **F**, Poxviridae). Black boxes indicate cells with biases opposite of the expected and with a frequency of greater than 1.05 for either observed or expected ratio.

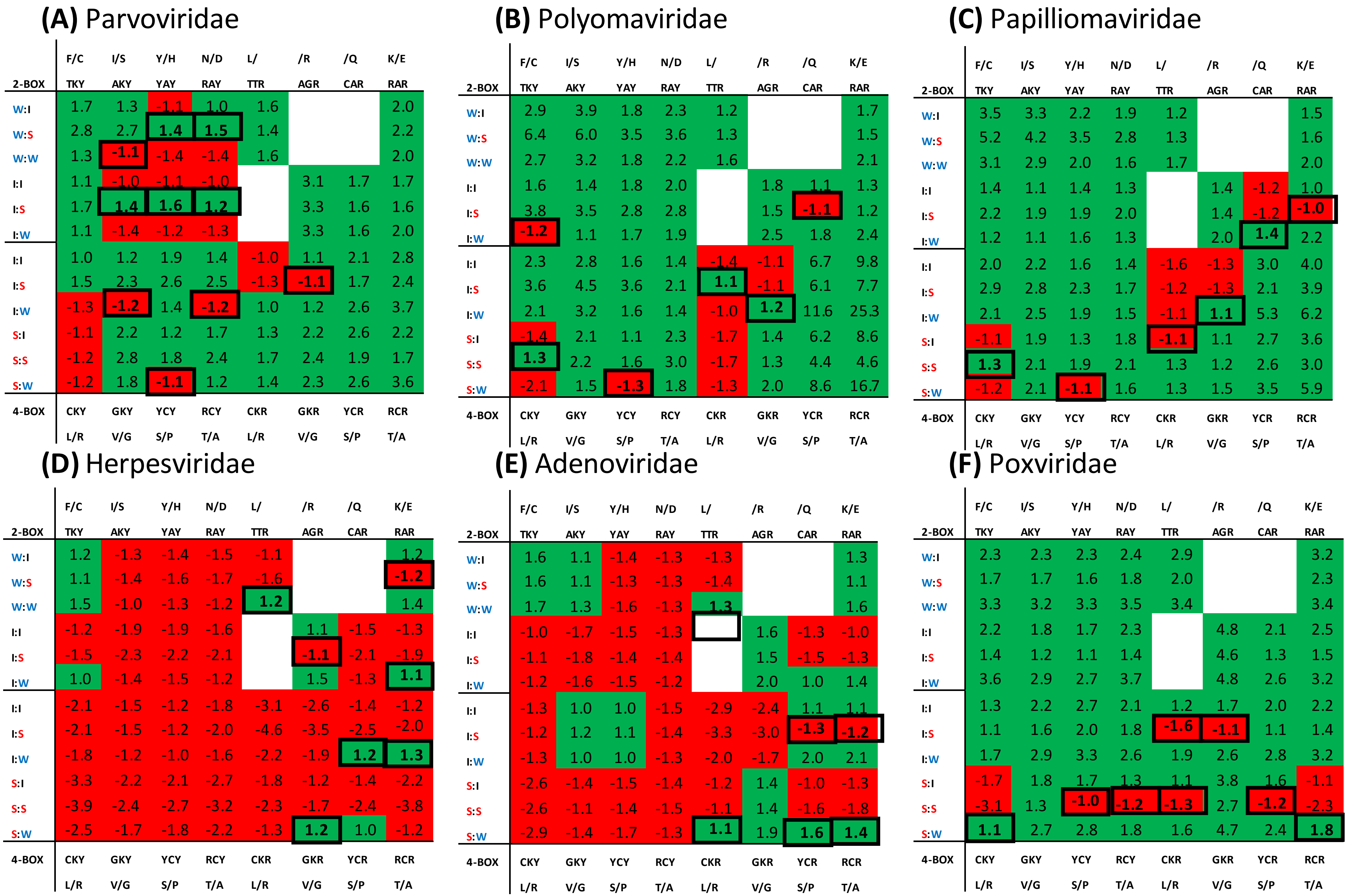
(A) Parvoviridae
F/C I/S Y/H N/D L/ /R /Q K/E
2-BOX TKY AKY YAY RAY TTR AGR CAR RAR
W:I 1.7 1.3 -1.1 1.0 1.6 2.0
W:S 2.8 2.7 1.4 1.5 1.4 2.2
W:W 1.3 -1.1 -1.4 -1.4 1.6 2.0
I:I 1.1 -1.0 -1.1 -1.0 3.1 1.7 1.7
I:S 1.7 1.4 1.6 1.2 3.3 1.6 1.6
I:W 1.1 -1.4 -1.2 -1.3 3.3 1.6 2.0
I:I 1.0 1.2 1.9 1.4 -1.0 1.1 2.1 2.8
I:S 1.5 2.3 2.6 2.5 -1.3 -1.1 1.7 2.4
I:W -1.3 -1.2 1.4 -1.2 1.0 1.2 2.6 3.7
S:I -1.1 2.2 1.2 1.7 1.3 2.2 2.6 2.2
S:S -1.2 2.8 1.8 2.4 1.7 2.4 1.9 1.7
S:W -1.2 1.8 -1.1 1.2 1.4 2.3 2.6 3.6
4-BOX CKY GKY YCY RCY CKR GKR YCR RCR
L/R V/G S/P T/A L/R V/G S/P T/A
(B) Polyomaviridae
F/C I/S Y/H N/D L/ /R /Q K/E
2-BOX TKY AKY YAY RAY TTR AGR CAR RAR
W:I 2.9 3.9 1.8 2.3 1.2 1.7
W:S 6.4 6.0 3.5 3.6 1.3 1.5
W:W 2.7 3.2 1.8 2.2 1.6 2.1
I:I 1.6 1.4 1.8 2.0 1.8 1.1 1.3
I:S 3.8 3.5 2.8 2.8 1.5 -1.1 1.2
I:W -1.2 1.1 1.7 1.9 2.5 1.8 2.4
I:I 2.3 2.8 1.6 1.4 -1.4 -1.1 6.7 9.8
I:S 3.6 4.5 3.6 2.1 1.1 -1.1 6.1 7.7
I:W 2.1 3.2 1.6 1.4 -1.0 1.2 11.6 25.3
S:I -1.4 2.1 1.1 2.3 -1.7 1.4 6.2 8.6
S:S 1.3 2.2 1.6 3.0 -1.7 1.3 4.4 4.6
S:W -2.1 1.5 -1.3 1.8 -1.3 2.0 8.6 16.7
4-BOX CKY GKY YCY RCY CKR GKR YCR RCR
L/R V/G S/P T/A L/R V/G S/P T/A
(C) Papilliomaviridae
F/C I/S Y/H N/D L/ /R /Q K/E
2-BOX TKY AKY YAY RAY TTR AGR CAR RAR
W:I 3.5 3.3 2.2 1.9 1.2 1.5
W:S 5.2 4.2 3.5 2.8 1.3 1.6
W:W 3.1 2.9 2.0 1.6 1.7 2.0
I:I 1.4 1.1 1.4 1.3 1.4 -1.2 1.0
I:S 2.2 1.9 1.9 2.0 1.4 -1.2 -1.0
I:W 1.2 1.1 1.6 1.3 2.0 1.4 2.2
I:I 2.0 2.2 1.6 1.4 -1.6 -1.3 3.0 4.0
I:S 2.9 2.8 2.3 1.7 -1.2 -1.3 2.1 3.9
I:W 2.1 2.5 1.9 1.5 -1.1 1.1 5.3 6.2
S:I -1.1 1.9 1.3 1.8 -1.1 1.1 2.7 3.6
S:S 1.3 2.1 1.9 2.1 1.3 1.2 2.6 3.0
S:W -1.2 2.1 -1.1 1.6 1.3 1.5 3.5 5.9
4-BOX CKY GKY YCY RCY CKR GKR YCR RCR
L/R V/G S/P T/A L/R V/G S/P T/A
(D) Herpesviridae
F/C I/S Y/H N/D L/ /R /Q K/E
2-BOX TKY AKY YAY RAY TTR AGR CAR RAR
W:I 1.2 -1.3 -1.4 -1.5 -1.1 1.2
W:S 1.1 -1.4 -1.6 -1.7 -1.6 -1.2
W:W 1.5 -1.0 -1.3 -1.2 1.2 1.4
I:I -1.2 -1.9 -1.9 -1.6 1.1 -1.5 -1.3
I:S -1.5 -2.3 -2.2 -2.1 -1.1 -2.1 -1.9
I:W 1.0 -1.4 -1.5 -1.2 1.5 -1.3 1.1
I:I -2.1 -1.5 -1.2 -1.8 -3.1 -2.6 -1.4 -1.2
I:S -2.1 -1.5 -1.2 -2.0 -4.6 -3.5 -2.5 -2.0
I:W -1.8 -1.2 -1.0 -1.6 -2.2 -1.9 1.2 1.3
S:I -3.3 -2.2 -2.1 -2.7 -1.8 -1.2 -1.4 -2.2
S:S -3.9 -2.4 -2.7 -3.2 -2.3 -1.7 -2.4 -3.8
S:W -2.5 -1.7 -1.8 -2.2 -1.3 1.2 1.0 -1.2
4-BOX CKY GKY YCY RCY CKR GKR YCR RCR
L/R V/G S/P T/A L/R V/G S/P T/A
(E) Adenoviridae
F/C I/S Y/H N/D L/ /R /Q K/E
2-BOX TKY AKY YAY RAY TTR AGR CAR RAR
W:I 1.6 1.1 -1.4 -1.3 -1.3 1.3
W:S 1.6 1.1 -1.3 -1.3 -1.4 1.1
W:W 1.7 1.3 -1.6 -1.3 1.3 1.6
I:I -1.0 -1.7 -1.5 -1.3 1.6 -1.3 -1.0
I:S -1.1 -1.8 -1.4 -1.4 1.5 -1.5 -1.3
I:W -1.2 -1.6 -1.5 -1.2 2.0 1.0 1.4
I:I -1.3 1.0 1.0 -1.5 -2.9 -2.4 1.1 1.1
I:S -1.2 1.2 1.1 -1.4 -3.3 -3.0 -1.3 -1.2
I:W -1.3 1.0 1.0 -1.3 -2.0 -1.7 2.0 2.1
S:I -2.6 -1.4 -1.5 -1.4 -1.2 1.4 -1.0 -1.3
S:S -2.6 -1.1 -1.4 -1.5 -1.1 1.4 -1.6 -1.8
S:W -2.9 -1.4 -1.7 -1.3 1.1 1.9 1.6 1.4
4-BOX CKY GKY YCY RCY CKR GKR YCR RCR
L/R V/G S/P T/A L/R V/G S/P T/A
(F) Poxviridae
F/C I/S Y/H N/D L/ /R /Q K/E
2-BOX TKY AKY YAY RAY TTR AGR CAR RAR
W:I 2.3 2.3 2.3 2.4 2.9 3.2
W:S 1.7 1.7 1.6 1.8 2.0 2.3
W:W 3.3 3.2 3.3 3.5 3.4 3.4
I:I 2.2 1.8 1.7 2.3 4.8 2.1 2.5
I:S 1.4 1.2 1.1 1.4 4.6 1.3 1.5
I:W 3.6 2.9 2.7 3.7 4.8 2.6 3.2
I:I 1.3 2.2 2.7 2.1 1.2 1.7 2.0 2.2
I:S 1.1 1.6 2.0 1.8 -1.6 -1.1 1.1 1.4
I:W 1.7 2.9 3.3 2.6 1.9 2.6 2.8 3.2
S:I -1.7 1.8 1.7 1.3 1.1 3.8 1.6 -1.1
S:S -3.1 1.3 -1.0 -1.2 -1.3 2.7 -1.2 -2.3
S:W 1.1 2.7 2.8 1.8 1.6 4.7 2.4 1.8
4-BOX CKY GKY YCY RCY CKR GKR YCR RCR
L/R V/G S/P T/A L/R V/G S/P T/A

Supplementary Figure 5. **Bias at the third nucleotide of codon pairs in virus and host genomes.** **Plant & Ye, 2022**

Heatmaps for (**A**) pig and (**B**) mouse. These heatmaps complement the heatmaps from more diverse vertebrate shown in Figure 5 of the manuscript. Black boxes indicate cells with biases opposite of the expected and with a frequency of greater than 1.05 for either observed or expected ratio.

(A) *Sus scrofa*

	F/C	I/S	Y/H	N/D	L/	/R	/Q	K/E
2-BOX	TKY	AKY	YAY	RAY	TTR	AGR	CAR	RAR
W:I	-1.2	-1.3	-1.3	-1.2	-1.6			-1.3
W:S	1.4	1.1	1.1	1.3	-1.9			-1.2
W:W	-1.6	-1.8	-1.7	-1.6	-1.3			-1.4
I:I	-1.2	-1.7	-1.5	-1.2		-1.0	-3.0	-1.4
I:S	1.3	-1.1	-1.1	1.2		1.0	-2.9	-1.5
I:W	-1.8	-2.1	-1.9	-1.6		-1.0	-3.2	-1.2
I:I	-1.4	-1.3	-1.2	-1.5	-6.2	-4.3	2.4	1.9
I:S	1.2	1.3	1.5	1.2	-7.4	-4.6	1.9	1.8
I:W	-2.5	-2.0	-2.2	-2.7	-4.3	-3.1	3.8	2.7
S:I	-2.4	-1.2	-2.2	-1.6	-1.8	-1.0	2.1	2.0
S:S	-1.5	1.4	-1.4	1.1	-2.2	-1.1	1.8	1.6
S:W	-3.3	-2.1	-3.3	-2.9	-1.5	1.2	3.0	3.2
4-BOX	CKY	GKY	YCY	RCY	CKR	GKR	YCR	RCR
	L/R	V/G	S/P	T/A	L/R	V/G	S/P	T/A

(B) *Mus musculus*

	F/C	I/S	Y/H	N/D	L/	/R	/Q	K/E
2-BOX	TKY	AKY	YAY	RAY	TTR	AGR	CAR	RAR
W:I	-1.2	-1.3	-1.3	-1.3	-1.9			-1.4
W:S	1.5	1.2	1.3	1.4	-2.0			-1.2
W:W	-2.0	-2.2	-2.0	-1.9	-1.5			-1.7
I:I	-1.0	-1.5	-1.4	-1.2		1.0	-2.8	-1.4
I:S	1.8	1.1	1.2	1.5		1.1	-2.4	-1.3
I:W	-1.8	-2.4	-2.0	-2.0		-1.2	-3.5	-1.5
I:I	-1.4	-1.3	-1.0	-1.2	-4.7	-3.7	3.0	2.9
I:S	1.5	1.4	2.0	1.6	-4.8	-3.4	3.0	3.1
I:W	-2.7	-2.3	-2.2	-2.7	-4.2	-3.4	3.6	2.8
S:I	-1.9	1.1	-1.8	-1.2	-1.5	1.1	3.1	3.0
S:S	1.0	2.2	1.0	1.6	-1.5	1.2	3.1	2.6
S:W	-3.3	-2.0	-3.3	-2.7	-1.6	1.0	3.2	3.2
4-BOX	CKY	GKY	YCY	RCY	CKR	GKR	YCR	RCR
	L/R	V/G	S/P	T/A	L/R	V/G	S/P	T/A

(A) Flaviviridae

	F/C	I/S	Y/H	N/D	L/	/R	/Q	K/E
2-BOX	TKY	AKY	YAY	RAY	TTR	AGR	CAR	RAR
W:I	-1.1	-1.2	-1.4	-1.3	-2.9			-1.1
W:S	-1.1	-1.2	-1.4	-1.3	-2.9			-1.1
W:W	-1.1	-1.2	-1.4	-1.3	-2.9			-1.1
I:I	-1.1	-1.2	-1.1	-1.3		1.1	-1.1	-1.0
I:S	-1.1	-1.2	-1.1	-1.3		1.1	-1.1	-1.0
I:W	-1.1	-1.2	-1.1	-1.3		1.1	-1.1	-1.0
I:I	-1.2	-1.1	-1.0	-1.2	-2.0	-3.4	2.2	1.9
I:S	-1.2	-1.1	-1.0	-1.2	-2.0	-3.4	2.2	1.9
I:W	-1.2	-1.1	-1.0	-1.2	-2.0	-3.4	2.2	1.9
S:I	-1.3	1.0	-1.2	1.0	-1.2	1.6	2.3	1.6
S:S	-1.3	1.0	-1.2	1.0	-1.2	1.6	2.3	1.6
S:W	-1.3	1.0	-1.2	1.0	-1.2	1.6	2.3	1.6
4-BOX	CKY	GKY	YCY	RCY	CKR	GKR	YCR	RCR
	L/R	V/G	S/P	T/A	L/R	V/G	S/P	T/A

(B) Yellow fever virus

	F/C	I/S	Y/H	N/D	L/	/R	/Q	K/E
2-BOX	TKY	AKY	YAY	RAY	TTR	AGR	CAR	RAR
W:I	1.2	-1.1	-1.3	-1.2	-9.0			-1.1
W:S	1.2	-1.1	-1.3	-1.2	-9.0			-1.1
W:W	1.2	-1.1	-1.3	-1.2	-9.0			-1.1
I:I	-1.0	1.1	1.6	-1.3		-1.2	-1.2	1.1
I:S	-1.0	1.1	1.6	-1.3		-1.2	-1.2	1.1
I:W	-1.0	1.1	1.6	-1.3		-1.2	-1.2	1.1
I:I	-1.1	-1.0	1.0	1.0	-2.7	-8.3	7.0	3.5
I:S	-1.1	-1.0	1.0	1.0	-2.7	-8.3	7.0	3.5
I:W	-1.1	-1.0	1.0	1.0	-2.7	-8.3	7.0	3.5
S:I	-2.1	1.4	-1.9	1.0	1.1	1.7	4.7	2.5
S:S	-2.1	1.4	-1.9	1.0	1.1	1.7	4.7	2.5
S:W	-2.1	1.4	-1.9	1.0	1.1	1.7	4.7	2.5
4-BOX	CKY	GKY	YCY	RCY	CKR	GKR	YCR	RCR
	L/R	V/G	S/P	T/A	L/R	V/G	S/P	T/A

(C) Dengue virus

	F/C	I/S	Y/H	N/D	L/	/R	/Q	K/E
2-BOX	TKY	AKY	YAY	RAY	TTR	AGR	CAR	RAR
W:I	1.0	1.0	-1.2	-1.4	-1.6			1.7
W:S	1.0	1.0	-1.2	-1.4	-1.6			1.7
W:W	1.0	1.0	-1.2	-1.4	-1.6			1.7
I:I	-1.0	-1.2	-1.0	-1.4		2.2	1.4	1.7
I:S	-1.0	-1.2	-1.0	-1.4		2.2	1.4	1.7
I:W	-1.0	-1.2	-1.0	-1.4		2.2	1.4	1.7
I:I	-1.3	-1.1	1.1	-1.2	-1.3	-3.0	6.2	3.9
I:S	-1.3	-1.1	1.1	-1.2	-1.3	-3.0	6.2	3.9
I:W	-1.3	-1.1	1.1	-1.2	-1.3	-3.0	6.2	3.9
S:I	-1.3	-1.2	-1.2	-1.3	1.7	2.8	7.0	3.5
S:S	-1.3	-1.2	-1.2	-1.3	1.7	2.8	7.0	3.5
S:W	-1.3	-1.2	-1.2	-1.3	1.7	2.8	7.0	3.5
4-BOX	CKY	GKY	YCY	RCY	CKR	GKR	YCR	RCR
	L/R	V/G	S/P	T/A	L/R	V/G	S/P	T/A

Supplementary Figure 6. Plant & Ye, 2022

Expected wobble position bias differs among related viruses. Data for the Flaviviridae family (**A**), two flaviviruses that infect humans (**B**, Dengue and **C**, Yellow fever virus), and two insect specific viruses (**D**, Aedes flavivirus and **E**, Culex flavivirus) are shown. Codon-pair frequency data is more limited when single viral genomes are used rather than families. .

(D) Aedes flavivirus

	F/C	I/S	Y/H	N/D	L/	/R	/Q	K/E
2-BOX	TKY	AKY	YAY	RAY	TTR	AGR	CAR	RAR
W:I	1.2	1.2	-1.1	-1.3	-2.5			1.6
W:S	1.2	1.2	-1.1	-1.3	-2.5			1.6
W:W	1.2	1.2	-1.1	-1.3	-2.5			1.6
I:I	1.1	-1.2	1.3	-1.9		1.3	1.7	1.2
I:S	1.1	-1.2	1.3	-1.9		1.3	1.7	1.2
I:W	1.1	-1.2	1.3	-1.9		1.3	1.7	1.2
I:I	-1.7	1.2	-1.3	-1.0	-1.5	-1.9	-1.1	-1.0
I:S	-1.7	1.2	-1.3	-1.0	-1.5	-1.9	-1.1	-1.0
I:W	-1.7	1.2	-1.3	-1.0	-1.5	-1.9	-1.1	-1.0
S:I	1.0	1.1	-1.2	-1.3	1.2	1.6	1.6	1.3
S:S	1.0	1.1	-1.2	-1.3	1.2	1.6	1.6	1.3
S:W	1.0	1.1	-1.2	-1.3	1.2	1.6	1.6	1.3
4-BOX	CKY	GKY	YCY	RCY	CKR	GKR	YCR	RCR

(E) Culex flavivirus

	F/C	I/S	Y/H	N/D	L/	/R	/Q	K/E
2-BOX	TKY	AKY	YAY	RAY	TTR	AGR	CAR	RAR
W:I	-1.5	-1.4	-3.1	-2.1	-4.7			-2.3
W:S	-1.5	-1.4	-3.1	-2.1	-4.7			-2.3
W:W	-1.5	-1.4	-3.1	-2.1	-4.7			-2.3
I:I	1.1	-2.0	-2.0	-1.9		-2.3	-1.4	-1.2
I:S	1.1	-2.0	-2.0	-1.9		-2.3	-1.4	-1.2
I:W	1.1	-2.0	-2.0	-1.9		-2.3	-1.4	-1.2
I:I	-1.6	1.3	-1.0	-1.3	-2.1	-10.8	1.4	-1.2
I:S	-1.6	1.3	-1.0	-1.3	-2.1	-10.8	1.4	-1.2
I:W	-1.6	1.3	-1.0	-1.3	-2.1	-10.8	1.4	-1.2
S:I	-1.3	-1.2	1.2	1.7	-1.2	2.1	-1.3	-1.3
S:S	-1.3	-1.2	1.2	1.7	-1.2	2.1	-1.3	-1.3
S:W	-1.3	-1.2	1.2	1.7	-1.2	2.1	-1.3	-1.3
4-BOX	CKY	GKY	YCY	RCY	CKR	GKR	YCR	RCR

Supplementary Figure 7. Bias at the third nucleotide of codon pairs in virus and host genomes. Plant & Ye, 2022

Heatmaps for many virus and host ORFs do not closely match each other. Heatmaps for **(A)** Escherichia coli and **(B-C)** two phages (T4 and phiX174), **(D)** Saccharomyces cerevisiae and **(E)** virus L-A, and **(F)** Arabidopsis thaliana and **(G)** Cauliflower Mosaic Virus are shown. Black boxes indicate cells with biases opposite of the expected and with a frequency of greater than 1.05 for either observed or expected ratio.

(A) *Escherichia coli*

	F/C	I/S	Y/H	N/D	L/	/R	/Q	K/E
2-BOX	TKY	AKY	YAY	RAY	TTR	AGR	CAR	RAR
W:I	1.2	1.1	1.3	-1.3	1.0			3.3
W:S	1.4	1.3	1.3	-1.3	1.1			3.3
W:W	1.9	1.3	1.5	**1.1**	-1.0			2.8
I:I	-1.3	-1.9	1.3	1.4		1.5	-1.9	2.4
I:S	-1.4	-2.0	1.2	1.6		1.4	-2.7	2.4
I:W	**1.1**	-1.3	1.7	2.3		2.0	-1.4	1.7
I:I	1.1	1.1	-1.0	-2.6	-13.3	-2.3	-1.2	-2.0
I:S	1.0	1.3	-1.1	-2.5	-16.5	-2.5	-1.1	-2.0
I:W	1.1	1.4	**1.2**	-2.4	-10.0	-2.3	-1.1	-1.3
S:I	-1.2	1.3	-1.3	-1.8	-1.7	-1.4	-2.9	-1.6
S:S	-1.1	1.3	-1.3	-1.5	-1.8	-1.6	-2.4	-1.6
S:W	**1.2**	1.2	1.0	-1.4	-1.2	-1.1	-2.5	-1.5
4-BOX	CKY	GKY	YCY	RCY	CKR	GKR	YCR	RCR
	L/R	V/G	S/P	T/A	L/R	V/G	S/P	T/A

(B) Phage T4

	F/C	I/S	Y/H	N/D	L/	/R	/Q	K/E
2-BOX	TKY	AKY	YAY	RAY	TTR	AGR	CAR	RAR
W:I	3.2	4.8	3.3	2.6	2.5			4.1
W:S	2.4	5.2	4.2	3.1	2.6			4.0
W:W	3.1	4.0	3.3	3.1	2.8			3.1
I:I	1.8	1.9	3.4	3.1		3.9	1.9	5.5
I:S	1.7	1.3	2.8	2.1		6.0	2.4	5.7
I:W	2.4	2.3	4.0	4.2		8.6	1.8	5.6
I:I	4.8	6.0	6.5	3.9	1.3	3.5	4.2	3.3
I:S	4.2	5.3	5.4	4.7	1.4	4.5	4.1	3.7
I:W	4.3	5.9	9.5	5.2	1.1	2.7	6.4	2.8
S:I	3.4	11.3	3.4	6.1	3.2	3.4	2.7	3.2
S:S	4.1	10.3	2.7	5.2	12.8	3.5	2.2	2.7
S:W	2.8	21.5	4.2	6.1	6.4	8.7	3.6	3.0
4-BOX	CKY	GKY	YCY	RCY	CKR	GKR	YCR	RCR
	L/R	V/G	S/P	T/A	L/R	V/G	S/P	T/A

(C) Phage phiX174

	F/C	I/S	Y/H	N/D	L/	/R	/Q	K/E
2-BOX	TKY	AKY	YAY	RAY	TTR	AGR	CAR	RAR
W:I	1.9	3.8	1.7	2.3	-1.9			**-1.1**
W:S	2.4	2.9	4.3	2.2	-1.9			4.2
W:W	1.7	3.5	6.3	**-1.1**	-1.0			2.2
I:I	**1.4**	**-2.5**	4.0	1.0		2.5	-1.0	-1.6
I:S	-2.0	**-1.0**	2.0	**-1.5**			-1.1	-1.7
I:W	-1.5	5.5	2.7	2.9		9.0	-1.9	-2.0
I:I	3.0	6.8	2.7	2.4	-3.5	-2.0	**1.4**	-2.6
I:S	4.8	5.7	10.5	8.5	-4.0	**1.1**	-2.0	-1.0
I:W	1.9	3.1	2.4	1.5	-2.0	-2.0	-1.6	-1.1
S:I	1.4	9.0	1.9	2.5	**-1.7**	6.0	-3.2	-1.7
S:S	1.3	7.0	1.4	4.4	4.0		-6.0	-1.3
S:W	**-1.0**	1.7	1.7	12.0	1.3	3.3	-1.0	**1.5**
4-BOX	CKY	GKY	YCY	RCY	CKR	GKR	YCR	RCR
	L/R	V/G	S/P	T/A	L/R	V/G	S/P	T/A

Supplementary Figure 7. Plant & Ye, 2022 Heatmaps for many virus and host ORFs do not closely match each other. Heatmaps for **(A)** Escherichia coli and **(B-C)** two phages (T4 and phiX174), **(D)** Saccharomyces cerevisiae and **(E)** virus L-A, and **(F)** Arabidopsis thaliana and **(G)** Cauliflower Mosaic Virus are shown. Black boxes indicate cells with biases opposite of the expected and with a frequency of greater than 1.05 for either observed or expected ratio.

(D) *Saccharomyces cerevisiae*

	F/C	I/S	Y/H	N/D	L/	/R	/Q	K/E
2-BOX	TKY	AKY	YAY	RAY	TTR	AGR	CAR	RAR
W:I	1.3	1.5	1.4	1.3	-1.6			1.1
W:S	2.1	2.0	1.9	1.8	-1.1			1.6
W:W	-1.7	-1.4	-1.4	-1.4	-2.2			-1.8
I:I	1.6	1.4	1.9	2.5		1.9	1.4	1.3
I:S	2.5	2.1	2.3	3.2		2.7	2.0	2.0
I:W	-1.1	-1.1	1.2	1.5		1.3	-1.2	-1.5
I:I	1.9	2.5	2.8	2.1	1.0	-1.5	2.3	2.4
I:S	2.5	3.4	3.5	2.4	1.4	-1.1	2.6	2.3
I:W	-1.1	1.4	1.4	1.2	-1.3	-2.1	1.8	2.3
S:I	2.4	4.2	2.5	3.5	1.4	2.6	2.1	2.3
S:S	3.1	5.6	3.5	4.2	2.2	4.7	2.3	2.7
S:W	1.7	2.2	1.8	1.8	-1.2	1.5	2.2	2.0
4-BOX	CKY	GKY	YCY	RCY	CKR	GKR	YCR	RCR
	L/R	V/G	S/P	T/A	L/R	V/G	S/P	T/A

(E) Virus L-A

	F/C	I/S	Y/H	N/D	L/	/R	/Q	K/E
2-BOX	TKY	AKY	YAY	RAY	TTR	AGR	CAR	RAR
W:I	1.3	2.0	-1.2	-1.2	1.4			-1.1
W:S	-1.5	-1.0	1.6	1.1	1.3			-1.1
W:W	-1.5	-1.6	-1.1	-1.9	-3.8			-1.3
I:I	7.0	3.6	1.3	1.7		1.0	1.6	1.8
I:S	-1.2	1.2	1.4	2.3		5.0	-1.4	2.4
I:W	1.5	1.3	-2.6	-1.2		-1.0	-1.3	4.0
I:I	1.3	1.3	6.0	3.1	-1.2	1.4	-2.1	1.9
I:S	-1.3	1.5		1.7	6.0	1.5	2.0	2.5
I:W	-1.5	1.4		6.2	-1.0	1.1	2.4	-1.2
S:I	-1.5	2.5	1.3	3.2	-1.2	2.1	3.7	3.3
S:S	2.0	1.2	1.3	2.0	2.5	1.8	3.8	1.8
S:W	1.3	-1.7	1.5	3.0	-2.0		6.0	-1.2
4-BOX	CKY	GKY	YCY	RCY	CKR	GKR	YCR	RCR
	L/R	V/G	S/P	T/A	L/R	V/G	S/P	T/A

(F) *Arabidopsis thaliana*

	F/C	I/S	Y/H	N/D	L/	/R	/Q	K/E
2-BOX	TKY	AKY	YAY	RAY	TTR	AGR	CAR	RAR
W:I	1.2	1.1	1.3	-1.3	1.0			3.3
W:S	1.4	1.3	1.3	-1.3	1.1			3.3
W:W	1.9	1.3	1.5	1.1	-1.0			2.8
I:I	-1.3	-1.9	1.3	1.4		1.5	-1.9	2.4
I:S	-1.4	-2.0	1.2	1.6		1.4	-2.7	2.4
I:W	1.1	-1.3	1.7	2.3		2.0	-1.4	1.7
I:I	1.1	1.1	-1.0	-2.6	-13.3	-2.3	-1.2	-2.0
I:S	1.0	1.3	-1.1	-2.5	-16.5	-2.5	-1.1	-2.0
I:W	1.1	1.4	1.2	-2.4	-10.0	-2.3	-1.1	-1.3
S:I	-1.2	1.3	-1.3	-1.8	-1.7	-1.4	-2.9	-1.6
S:S	-1.1	1.3	-1.3	-1.5	-1.8	-1.6	-2.4	-1.6
S:W	1.2	1.2	1.0	-1.4	-1.2	-1.1	-2.5	-1.5
4-BOX	CKY	GKY	YCY	RCY	CKR	GKR	YCR	RCR
	L/R	V/G	S/P	T/A	L/R	V/G	S/P	T/A

(G) Cauliflower Mosaic Virus

	F/C	I/S	Y/H	N/D	L/	/R	/Q	K/E
2-BOX	TKY	AKY	YAY	RAY	TTR	AGR	CAR	RAR
W:I	3.2	4.8	3.3	2.6	2.5			4.1
W:S	2.4	5.2	4.2	3.1	2.6			4.0
W:W	3.1	4.0	3.3	3.1	2.8			3.1
I:I	1.8	1.9	3.4	3.1		3.9	1.9	5.5
I:S	1.7	1.3	2.8	2.1		6.0	2.4	5.7
I:W	2.4	2.3	4.0	4.2		8.6	1.8	5.6
I:I	4.8	6.0	6.5	3.9	1.3	3.5	4.2	3.3
I:S	4.2	5.3	5.4	4.7	1.4	4.5	4.1	3.7
I:W	4.3	5.9	9.5	5.2	1.1	2.7	6.4	2.8
S:I	3.4	11.3	3.4	6.1	3.2	3.4	2.7	3.2
S:S	4.1	10.3	2.7	5.2	12.8	3.5	2.2	2.7
S:W	2.8	21.5	4.2	6.1	6.4	8.7	3.6	3.0
4-BOX	CKY	GKY	YCY	RCY	CKR	GKR	YCR	RCR
	L/R	V/G	S/P	T/A	L/R	V/G	S/P	T/A
